# Reduced structural rigidity of MDMX protein enhances binding to *TP53* mRNA

**DOI:** 10.1042/BSR20253646

**Published:** 2025-11-25

**Authors:** Martina Kucerikova, Ondrej Bonczek, Vanesa Olivares-Illana, Andres Rodriguez-Rodriguez, Jose G. Sampedro, Lenka Hernychova, Vaclav Hrabal, Pavlina Zatloukalova, Radovan Krejcir, Robin Fahraeus, Philip J. Coates, Borivoj Vojtesek, Lucia Martinkova

**Affiliations:** 1 RECAMO, Masaryk Memorial Cancer Institute, Brno, 602 00, Czechia; 2 National Centre for Biomolecular Research, Faculty of Science, Masaryk University, Brno, 625 00, Czechia; 3 Instituto de Fisica, Universidad Autonoma de San Luis Potosi, San Luis Potosi, 78290, Mexico; 4 Department of Experimental Biology, Faculty of Science, Masaryk University, Brno, 625 00, Czechia; 5 Inserm UMRS1131, Institut de Genetique Moleculaire, Universite Paris Cite, Hopital St. Louis, Paris, 75010, France; 6 Department of Medical Biosciences, Umea University, Umea, 901 87, Sweden; 7 Laboratory of Growth Regulators, Institute of Experimental Botany, The Czech Academy of Sciences, Olomouc, 779 00, Czechia

**Keywords:** HDX-MS mapping, MDMX–RNA interaction, RING domain, *TP53*mRNA

## Abstract

The two murine double minute (MDM) family members, MDM2 and MDMX, are a well-established negative regulator of p53 activity. Under DNA damage conditions, MDM2 and MDMX are phosphorylated near their RING domains (serine 395 at MDM2 and serine 403 at MDMX) and switch to act as p53 positive regulators. MDMX binds to *TP53* mRNA and acts as a chaperone for RNA structure, enabling MDM2 to bind. This interaction enhances *TP53* mRNA translation, leading to increased p53 protein production. While the biological significance of this interaction has been described, the specific features of the MDMX–RNA interaction remain poorly understood. We used various MDMX protein constructs to characterize binding to *TP53* mRNA and identified that the interaction mediated by the RING domain is modulated by the presence of other domains. Hydrogen-deuterium exchange mass spectrometry (HDX-MS) and binding assays in high salt conditions and various pH demonstrate that the whole protein participates in RNA interaction, with the C-terminal domain likely providing the contact with RNA by electrostatic forces. We show that protein structural changes induced by the chelating agent EDTA or the reducing agent TCEP enhance RNA binding by promoting partial structural destabilization of the protein. Our findings suggest that the MDMX/*TP53* mRNA interaction is complex, with the RING domain binding to RNA and being supported by the entire protein, which acts as a scaffold for the RNA interaction. These results contribute to a better understanding of MDMX’s role in *TP53* mRNA binding and provide valuable insights for future investigation of the MDM2–MDMX–*TP53* mRNA complex, which is crucial for p53 stabilization and activation under DNA-damaging conditions.

## Introduction

MDMX is an oncoprotein that is amplified and overexpressed in various cancers, leading to diminished p53 activity and promoting tumor growth and survival [1,2]. This aberrant expression is often mutually exclusive with *TP53* mutations and *MDM2* amplification and is frequently associated with poor prognosis in cancer patients, highlighting its crucial role in tumorigenesis [1,2]. Under normal conditions, MDMX, in collaboration with its homolog MDM2, plays a key role in maintaining low levels of p53 protein by directing p53 for proteasomal degradation [1]. MDMX also exerts p53-independent functions, including regulation of cell cycle, genomic instability, and metabolism [3–5]. During DNA damage, a phosphorylation cascade triggered by ataxia-telangiectasia mutated (ATM) kinase disrupts the p53–MDM2–MDMX interaction, resulting in increased p53 protein levels and activation [6]. ATM directly phosphorylates MDM2 at serine 395 and MDMX at serine 403. As a result, MDMX and MDM2 shift their function to act as positive regulators of p53 activity by directly binding *TP53* mRNA and enhancing its translation [7]. In this context, MDMX acts as an RNA chaperone protein for *TP53* mRNA and enables the efficient binding of MDM2 [8]. Recently, it was shown that the ATM kinase also binds *TP53* mRNA under stress conditions, and MDMX prevents this interaction and subsequent interaction with Nijmegen breakage syndrome 1 (NBS1), a component of the MRN (MRE11–RAD50–NBS1) complex involved in DNA damage recognition and repair [9].

**Figure 1:**
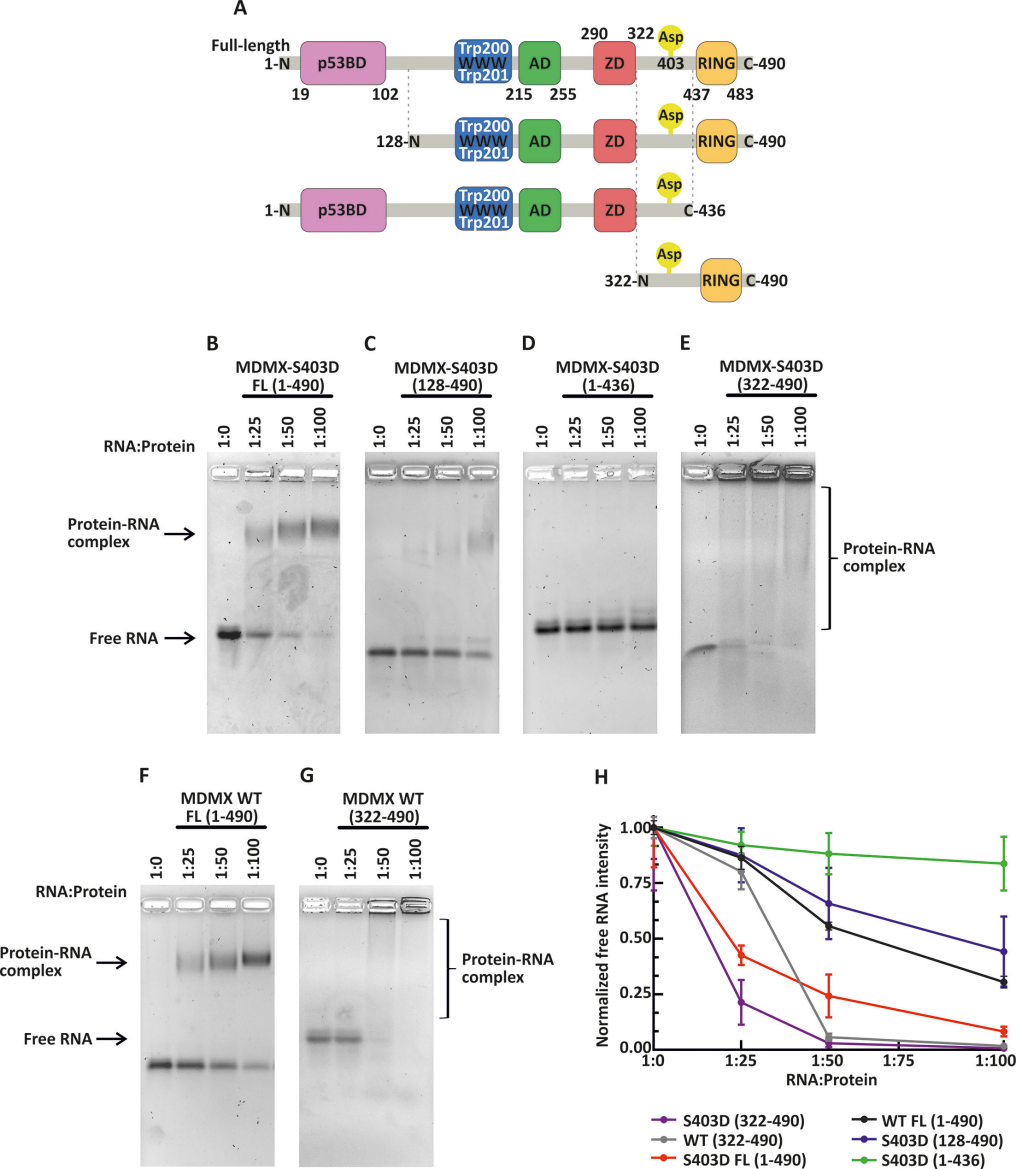
*TP53* mRNA binding to phosphomimetic and wildtype MDMX protein constructs. (**A**) Schematic representation of MDMX protein constructs carrying a phosphomimetic mutation at serine 403 (MDMX-S403D), shown in four variants: full-length MDMX-S403D FL (1–490), a truncated form lacking the N-terminal domain MDMX-S403D (128–490), a construct lacking the C-terminal region including the RING domain MDMX-S403D (1–436), and a C-terminal fragment containing only the RING domain MDMX-S403D (322–490). The domain architecture includes the p53-binding domain (p53BD, pink), WWW domain (blue), acidic domain (AD, green), zinc-binding domain (ZD, red), and the RING domain (yellow). (**B–E**) EMSA analysis of *TP53* mRNA binding to the four MDMX-S403D variants shown in (**A**). (**F, G**) EMSA analysis of *TP53* mRNA binding to wildtype MDMX constructs: (**F**) full-length MDMX WT FL (1–491) and (**G**) the C-terminal RING-containing fragment MDMX WT (322–490). All recombinant proteins were incubated with *in vitro* transcribed *TP53* mRNA (nucleotides 1–240) at RNA:protein molar ratios of 1:25, 1:50, and 1:100. Free RNA (ratio 1:0) served as control. Reactions were performed in binding buffer (150 mM NaCl, 50 mM Tris, 1 mM TCEP, 10 µM ZnSO₄, pH 7.5). RNA–protein complexes and free RNA were visualized on agarose gels by GelRed staining. Brackets indicate smears corresponding to protein–*TP53* mRNA complexes. (**H**) Quantification of free RNA band intensity from EMSA gels shown in (B–G). For each RNA:protein ratio (1:25, 1:50, 1:100), free RNA intensity was normalized to the RNA-only control (1:0). Colored curves represent individual constructs: (**B**) MDMX-S403D FL (1–490), red; (**C**) MDMX-S403D (128–490), blue; (**D**) MDMX-S403D (1–436), green; (**E**) MDMX-S403D (322–490), purple; (**F**) MDMX WT FL (1–490), black; (**G**) MDMX WT (322–490), gray. Data represent mean ± SEM (*n* = 3 biological replicates).

Structurally, MDMX is an intrinsically disordered protein with several organized domains, including the p53-binding domain at its N terminus, the WWW element, the acidic domain, the zinc-finger domain, and the RING domain (Figure 1A). The RING domain, located at the C-terminus, is critical for heterodimerization with MDM2 [10]. Recent *in cellulo* studies have also described that homodimerization of MDMX influences its binding to p53 [11]. The RNA-binding activity of MDMX has been mapped to its RING domain [8]. Due to its structural organization, MDMX exhibits significant flexibility [12]. Therefore, studies of domain functions, complemented by full-length protein analysis, can provide a more comprehensive understanding.

We characterized key aspects of the MDMX–RNA interaction, providing new insights into how MDMX modulates *TP53* mRNA and its potential roles. We confirmed that the RING domain is essential for *TP5*3 mRNA interaction, while other domains play modulatory roles likely due to structural organization. Binding at various pH and high salt conditions, together with HDX-MS, revealed that protein-RNA binding is partially driven by electrostatic forces at the protein’s C-terminus, but the entire protein participates in binding and stabilizes the interaction. Furthermore, we investigated how reducing TCEP and chelating conditions EDTA affect both the structural organization of MDMX and its *TP53* mRNA-binding capacity, with conformational destabilization enhancing RNA interaction. The role of MDMX as an RNA-binding protein adds an important dimension to its regulatory repertoire. A deeper understanding of these interactions could lead to novel therapeutic approaches in cancer treatment.

## Material and methods

### Cloning and purification of protein constructs

The original purification protocol [12] was modified as follows: cDNA of MDMX wildtype (WT) or S403D constructs were cloned into HIS-tagged vectors pET28B (Merck Millipore, Darmstadt, Germany) and pDEST17 (Thermo Fisher Scientific, Waltham, MA, U.S.A.). The Ser403-to-Asp mutation mimics ATM kinase-mediated phosphorylation at this site in response to DNA damage. The insoluble protein fraction was resuspended in guanidine buffer (6 M guanidine hydrochloride, 50 mM Tris [pH 8.0], 150 mM NaCl, 10 mM imidazole, 2 mM MgCl₂) and incubated at 37°C for 90 min, with Turbonuclease from *Serratia marcescens* (Merck Millipore) added during the final 15 min of incubation to remove residual nucleic acids. The Ni-NTA Agarose (Qiagen, Hilden, Germany) was washed with 0.1 M Tris (pH 8), 2 M NaBr, 0.5 M NaCl, and 10 mM 2-mercaptoethanol. Proteins were eluted from the washed resin using 5 ml (5 fractions of 1 ml) of elution buffer (50 mM Tris (pH 8), 300 mM Imidazole, 10 µM ZnSO4·7H2O). Eluted proteins were dialyzed overnight at 4°C in 50 mM Tris (pH 8), 150 mM NaCl, and 10 µM ZnSO₄. After dialysis, proteins were concentrated using 10 kDa MWCO Amicon Ultra-4 centrifugal filters (Merck Millipore).

### Sodium dodecyl sulfate-polyacrylamide gel electrophoresis (SDS-PAGE)

SDS-PAGE was performed as described previously [13]. A 300 ng of purified protein was loaded per lane for electrophoretic separation, and gels were stained with Coomassie brilliant blue for 30 min. Protein visualization was performed using ChemiDoc Imaging (Bio-Rad, Hercules, CA, U.S.A.).

### Electrophoretic mobility shift assay (EMSA)

*In vitro* transcription of *TP53* mRNA was performed using the mMESSAGE mMACHINE™ T7 Transcription Kit (Thermo Fisher Scientific) according to the manufacturer’s instructions. The linearized pcDNA3-p53 plasmid (encoding nucleotides 1–240) [8] was used as the DNA template. A total of 100 ng of RNA was mixed with purified protein (in a 10 µl reaction) at RNA:protein molar ratios specified in the figures, using binding buffer (150 mM NaCl, 50 mM Tris, 1 mM TCEP, 10 µM ZnSO₄, pH 7.5) along with additional conditions outlined in the figures. Samples were incubated at room temperature for 20 min before being loaded onto 1.2% agarose gels. The gels were poststained with GelRed nucleic acid stain (Biotium, Fremont, CA, U.S.A.) for 40 min. For protein detection following the GelRed staining, the same gels were fixed in 50% methanol and 7% acetic acid for 20 min and subsequently stained overnight with SYPRO™ Ruby protein gel stain (Thermo Fisher Scientific). Both gels were imaged using ChemiDoc Imaging (Bio-Rad). Densitometric analysis was performed for all EMSA gels, and the intensity of free RNA was normalized to the RNA-only control. Data represent mean ± SEM from three independent biological replicates (*n* = 3). Statistical significance was determined using two-tailed Student’s *t*-test, with the following annotations: *P<0.05, **P<0.01, ***P<0.001. In addition, some comparisons between specific conditions are indicated in the figures using alternative symbols: •*P*<0.05, ••*P*<0.01, •••*P*<0.001.

### Hydrogen-deuterium exchange mass spectrometry (HDX-MS)

HDX-MS of the HDMX-S403D protein was conducted in both ligand-free and ligand-bound conformations with *TP53* mRNA, following the experimental procedures described in [14,15]. Detailed methodology and HDX-MS data are available in the ProteomeXchange (PX) Consortium [16] via the Proteomics Identifications (PRIDE) [17] repository (Dataset identifier: PXD060074; Username: reviewer_pxd060074@ebi.ac.uk; Password: LyazwVQgkNEC).

### Circular dichroism (CD) spectroscopy

Far-UV CD spectra of MDMX-S403D FL and S403D (322-490) were recorded using a Jasco J-1500 Spectrometer (Jasco Inc., Easton, MD, U.S.A.) in a wavelength range of 180–280 nm. Measurements were performed at a protein concentration of 0.5 mg/ml in phosphate buffer (pH 7.5), using quartz cuvettes with a 0.1-cm path length.

### Intrinsic fluorescence

The emission fluorescence spectra of the proteins (at 0.5 mg/ml concentration) in phosphate buffer (pH 7.5) or binding buffer were recorded using an Agilent Technologies Cary Eclipse Fluorescence Spectrophotometer (Agilent Technologies, Santa Clara, CA, U.S.A.). Measurements were performed at room temperature in quartz cuvettes with a 1-cm path length. The excitation wavelength was set to 280 nm, and blank measurements (without protein) were recorded and subtracted from the experimental spectra.

## Results

### Domain-specific contributions to the RNA-binding activity of the protein

To characterize the RNA-binding properties of MDMX, we prepared recombinant proteins in both the WT form and a phosphomimetic mutation at serine 403 (S403D), which enhances affinity for *TP53* mRNA [8]. For the S403D mutant, we generated four MDMX variants: full-length (FL; 1–490), a truncated construct lacking the N-terminal domain (128–490), a construct lacking the C-terminal region including the RING domain (1–436), and a C-terminal fragment containing only the RING domain (322–490). In addition, two WT constructs were produced: full-length (FL; 1–490) and the RING-containing C-terminal variant (322–490) (Figure 1A). All recombinant proteins were analyzed using EMSA. The *TP53* mRNA fragment (1–240), corresponding to the first 240 nucleotides of the full-length p53 coding sequence, was synthesized by *in vitro* transcription. This region includes the internal ribosome entry site (IRES) for the p53/47 isoform and has previously been identified as the MDMX-interacting region [8]. Binding assays were performed using 100 ng of *TP53* mRNA (nucleotides 1–240), hereafter referred to as ‘RNA’, and increasing concentrations of recombinant MDMX proteins. The RNA-protein formation was indicated as a reduction of free RNA and for protein variants FL (Figure 1B and F) and truncation 128–490 (Figure 1C) as a shifted gel band. The C-terminal constructs 322–490 (Figure 1E) migrated poorly into the gel, often appearing as a smear or remaining trapped in the gel wells. This behavior is likely due to the higher pI and structural properties of the construct, although the possibility of aggregation cannot be entirely ruled out. Moreover, protein conformational changes may reduce RNA accessibility in complex, thereby hindering its detectability by staining under certain conditions. Therefore, we evaluated the reduction of free RNA rather than the signal intensity of the protein–RNA complex to compare the RNA-binding affinity of all protein variants (Figure 1H), as well as protein-RNA affinity under other conditions (shown in later figures). As expected, in the absence of protein (lane 1:0), only free RNA was detected, serving as a reference for the unbound RNA signal (Figure 1B–G). MDMX-S403D FL (Figure 1B) showed enhanced binding to *TP53* mRNA, as evidenced by a reduction in free RNA and the appearance of a shifted RNA–protein complex at a 1:25 RNA:protein molar ratio, with near-complete formation of the complex observed at 1:100. The S403D variant lacking the N-terminal domain, MDMX-S403D (128–490), exhibited reduced RNA binding (Figure 1C), suggesting that the N-terminal region modulates or facilitates the RNA interaction. The MDMX-S403D (1–436) variant, which lacks the RING domain, did not bind *TP53* mRNA at the tested concentrations (Figure 1D), indicating that the RING domain is essential for RNA interaction. In contrast, the C-terminal variant MDMX-S403D (322–490), containing the RING domain, reduced free RNA across all tested RNA:protein ratios, indicating higher affinity than full-length MDMX-S403D (Figure 1E), as previously shown by RNA-ELISA [8]. However, smear rather than discrete bands are visible as complexes, leaving open the possibility that protein aggregation contributes to the observed binding. We also compared RNA binding of the two WT forms of MDMX, full-length (1–490) (Figure 1F) and the RING-containing C-terminal variant (322–490) (Figure 1G) and confirmed previous findings [8] that WT proteins bind *TP53* mRNA less efficiently than their phosphomimetic counterparts (Figure 1B and E). Although potential aggregation of the C-terminal variant constructs may contribute to the observed binding pattern, the findings nonetheless suggest that the RING domain plays an important role in the MDMX–*TP53* mRNA interaction. The S403D phosphomimetic mutation enhances binding affinity, whereas other domains potentially mask or modulate the RNA-binding interface.

### Mapping the RNA interaction site of the MDMX protein

**Figure 2:**
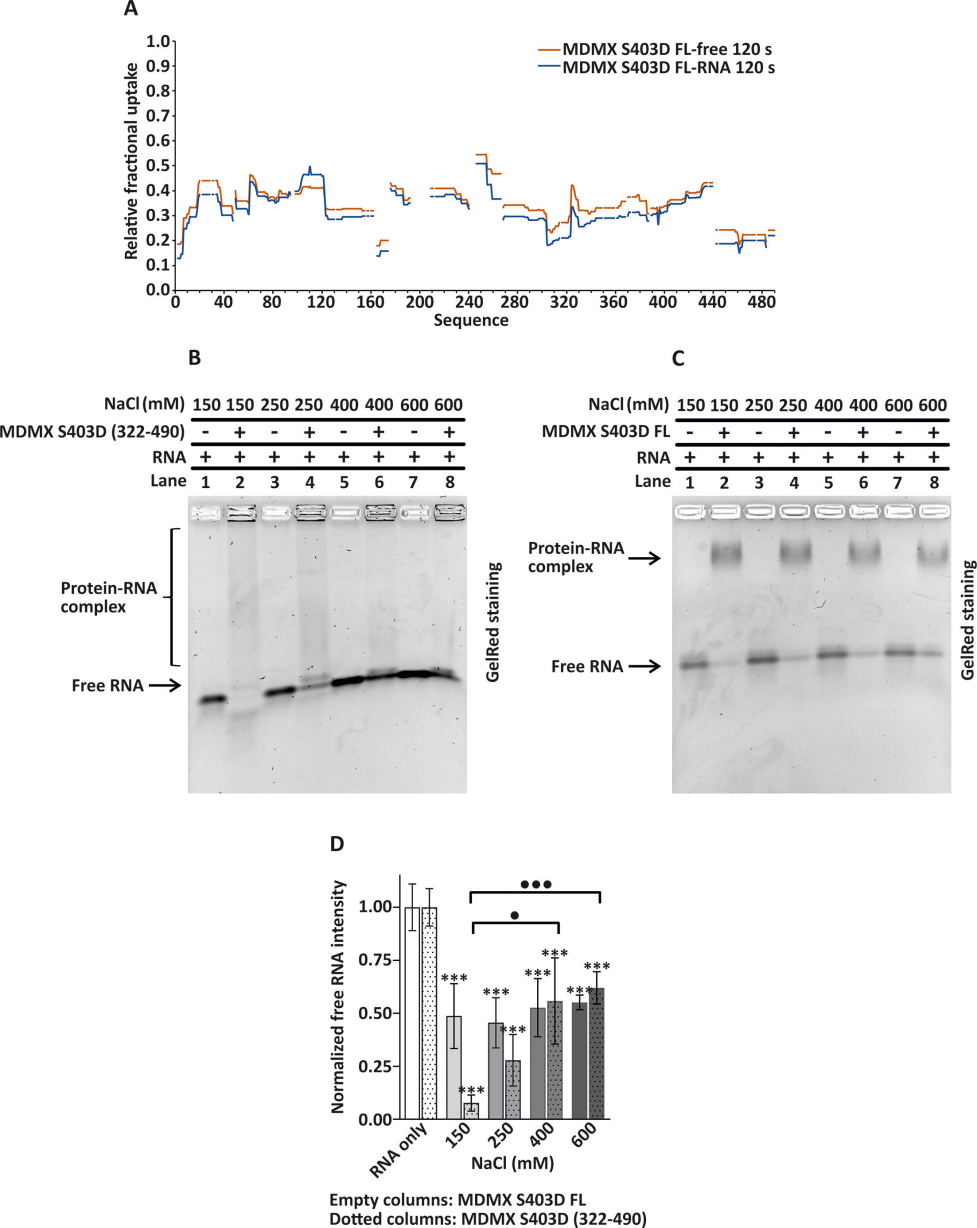
Characterization of the MDMX-S403D interaction with *TP53* mRNA by HDX-MS and EMSA under varying salt concentrations. (**A**) HDX-MS analysis of full-length MDMX carrying a phosphomimetic mutation at serine 403 (MDMX-S403D FL) in complex with *TP53* mRNA. Relative fractional uptake across amino acid residues is shown for free MDMX-S40A3D FL (orange) and the MDMX-S403D FL–*TP53* mRNA complex (blue) after 120 s deuterium exposure. Nonexchangeable proline residues are indicated as gaps in the profile. (**B, C**) EMSA analysis of *TP53* mRNA binding to (**B**) MDMX-S403D (322–490) and (**C**) MDMX-S403 FL under increasing NaCl concentrations (150, 250, 400, and 600 mM). Protein–RNA complexes (lanes 2, 4, 6, 8) and corresponding free RNA controls (lanes 1, 3, 5, 7) were visualized on agarose gels by GelRed at an RNA:protein molar ratio of 1:50. Brackets indicate smears corresponding to protein–*TP53* mRNA complexes. (**D**) Quantification of free RNA band intensity derived from the EMSA gels in (**B**) and (**C**). Data for MDMX-S403D FL are shown as empty columns (from gel C) and for MDMX-S403D (322–490) as dotted columns (from gel B), normalized to their respective RNA-only controls. Data represent mean ± SEM (*n* = 3 biological replicates; ***P<0.001). Additional statistical comparisons are shown in the figure and alternatively indicated as •*P*<0.05 and •••*P*<0.001.

MDMX is a highly flexible protein due to disordered regions between its structured domains [12] implying that certain protein or RNA interactions may exert allosteric effects that regulate its interactome [18]. To identify the RNA-binding site on MDMX-S403D FL (1-490) and to study its allosteric effect, we performed HDX-MS at three time intervals: 10 s (Supplementary Figure S1A), 120 s (Figure 2A), and 1 800 s (Supplementary Figure S1B). By this method, direct nucleic acid–protein interactions are seen as deuteration suppression, and rearrangements of protein structure due to ligand binding are seen as increased deuteration for structure opening or decreased deuteration for structure closing. Our results showed no specific RNA-binding site within the RING domain, but small deuteration changes were observed throughout the entire protein, with the most noticeable opening in the N-terminal domain (100–120 aa) (Figure 2A), supporting the notion that the entire protein participates, either directly or indirectly, in RNA binding. The RING domain (440–490) exhibits low deuteration (Figure 2A), suggesting masking by other protein domains. The inability to identify a direct RNA contact site may reflect limitations of the experimental settings, including restricted accessibility of the RING domain under the assay conditions, the potential absence of a well-defined binding interface, or the inherent instability, transient nature, and possible nonspecificity of the interaction, all of which could prevent reliable detection.

We used increasing salt concentrations in the binding assay to elucidate the nature of the interaction between MDMX and RNA. The C-terminal construct MDMX-S403D (322–490) reduced nearly all available RNA at 150 mM NaCl (Figure 2B, lane 2, quantified in Figure 2D) occurring along with the smear on the gel that suggests RNA–protein complex formation. However, as the salt concentration increased (starting at 250 mM NaCl), the amount of free RNA also increased, suggesting that higher salt concentration disrupted electrostatic interactions required for RNA–protein complex formation (Figure 2B, lanes 4, 6, and 8). In contrast, the RNA–protein complexes formed by MDMX-S403D FL were resilient to increasing salt concentrations (150–600 mM), remaining stable even at higher ionic strength (Figure 2C, quantified in Figure 2D). This suggests that the interaction is supported by additional, nonelectrostatic forces. Since salt concentration may influence the binding efficiency of GelRed stain to RNA (Supplementary Figure S2), each EMSA gel included free RNA controls loaded at the corresponding NaCl concentration. Altogether, these findings suggest that the C-terminal construct domain binds RNA via electrostatic forces that are not detectable by HDX-MS, while other MDMX domains may partially shield the RING interface and contribute to stabilization of the RNA–protein complex.

### The effect of pH on the binding of MDMX-S403D to RNA

**Figure 3:**
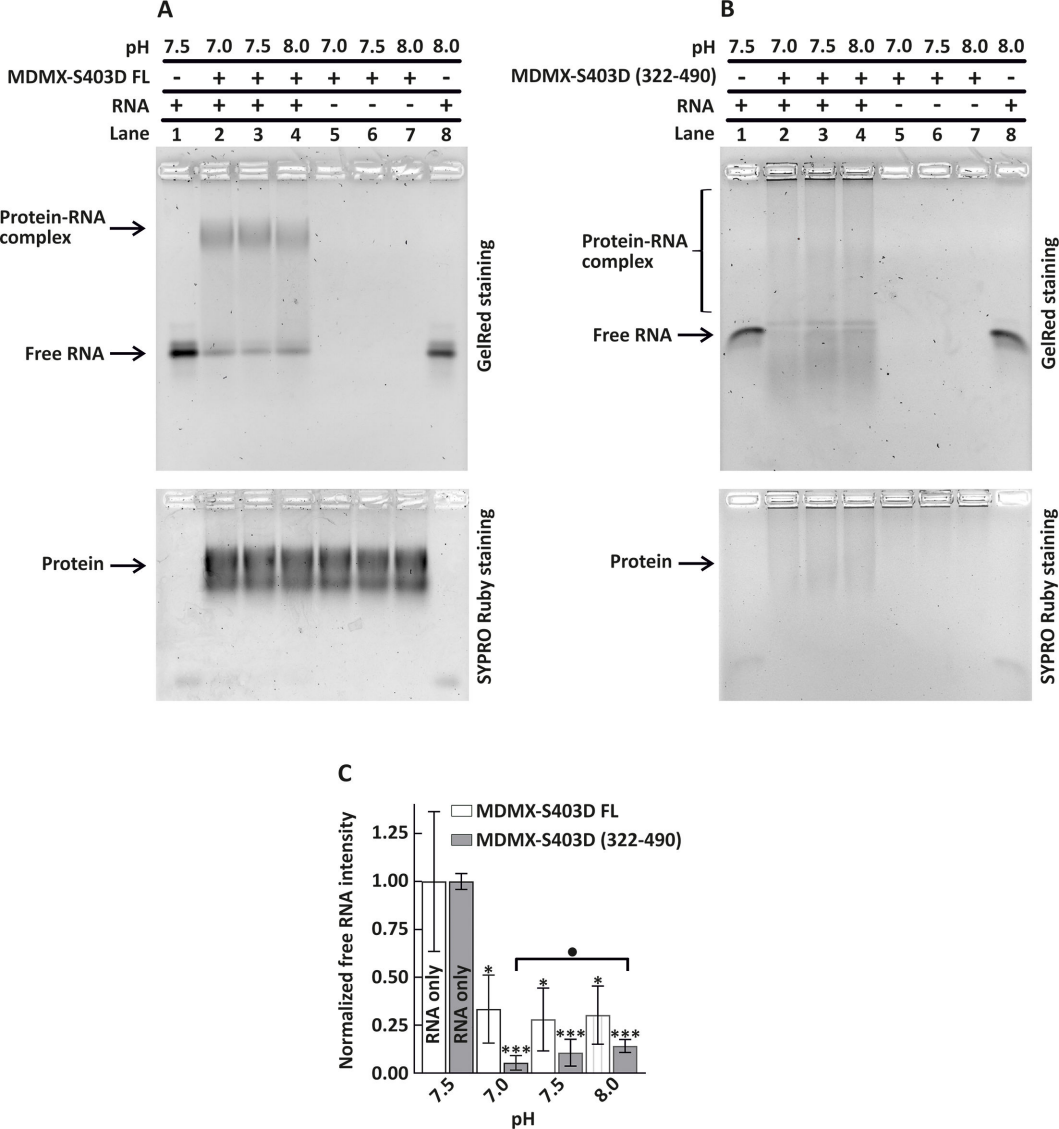
Effect of pH on *TP53* mRNA–MDMX interaction. (**A, B**) EMSA showing the effect of pH (7.0, 7.5, and 8.0) on *TP53* mRNA binding to (**A**) MDMX-S403D FL and (**B**) MDMX-S403D (322–490). Proteins were incubated with *TP53* mRNA (lanes 2–4) or without RNA (lanes 5–7) in binding buffer, adjusted to the indicated pH values. Free RNA controls were included at pH 7.5 and 8.0 (lanes 1 and 8). The RNA:protein molar ratio was 1:50 in all reactions. Brackets indicate smears corresponding to protein–TP53 mRNA complexes. RNA and RNA–protein complexes were visualized on GelRed-stained agarose gels (upper panels), and total protein was detected using SYPRO Ruby staining of the same gels (lower panels). (**C**) Quantification of free RNA band intensity derived from the EMSA gels in (**A**) and (**B**). Data for MDMX-S403 FL are shown as white columns (from gel A) and for MDMX-S403D (322–490) as gray columns (from gel B), normalized to their respective RNA-only control. Data represent mean ± SEM (*n* = 3 biological replicates; *P<0.05, ***P<0.001). Additional statistical comparisons are shown in the figure and alternatively indicated as •*P*<0.05.

To evaluate further the electrostatic contribution of protein–RNA interaction, we examined how the variations in pH (7.0, 7.5, and 8.0) will influence the RNA binding. The MDMX-S403D FL protein has an isoelectric point (pI) of 5.1, whereas the C-terminal construct MDMX-S403D (322–490) has a pI of 7.6. The pH shift from 7.0 to 8.0 primarily affects cysteine and histidine residues located mainly in the C-terminus region of MDMX, altering their protonation state and resulting in a more negative overall charge (Supplementary Figure S3) [19,20]. For the MDMX-S403D FL construct (Figure 3A, quantified in 3C), no significant difference in RNA binding was observed across the tested pH range (Figure 3A, lanes 2–4, upper panel). RNA alone exhibited a consistent migration pattern at pH 7.5 (lane 1) and pH 8.0 (lane 8). The MDMX-S403D (322–490) construct (Figure 3B, quantified in 3C) showed small but consistent reduction in RNA binding as pH increased (Figure 3B, lanes 2–4, upper panel), corresponding to reduced protonation at higher pH values leading to increased negative charge and weakened electrostatic interactions. To determine whether altered protein migration contributed to the observed effects, we stained the agarose gels with SYPRO Ruby (Figure 3A and B, lower panels). Across all tested pH conditions, the migration of both constructs remained unchanged, whether in free form (lanes 5–7) or as part of an RNA–protein complex (lanes 2–4), indicating that differences in EMSA results are not due to altered protein mobility. Moreover, these results show that the C-terminal truncation (322–490) alone penetrates poorly into the gel wells (Figure 3B, lower panel, lanes 5–7) and is only drawn efficiently into the gel in the presence of RNA (lane 2–4), appearing as a smear, whereas the full-length protein penetrates the gel more efficiently and forms discrete complexes visible as a band (Figure 3A, lower panel). These results reflect the electrostatic nature of RNA binding by the C-terminal region and highlight the role of the entire protein in stabilizing the RNA interaction across a broader pH range.

### Conditions influencing accessibility of the MDMX RING domain regulate RNA binding

**Figure 4:**
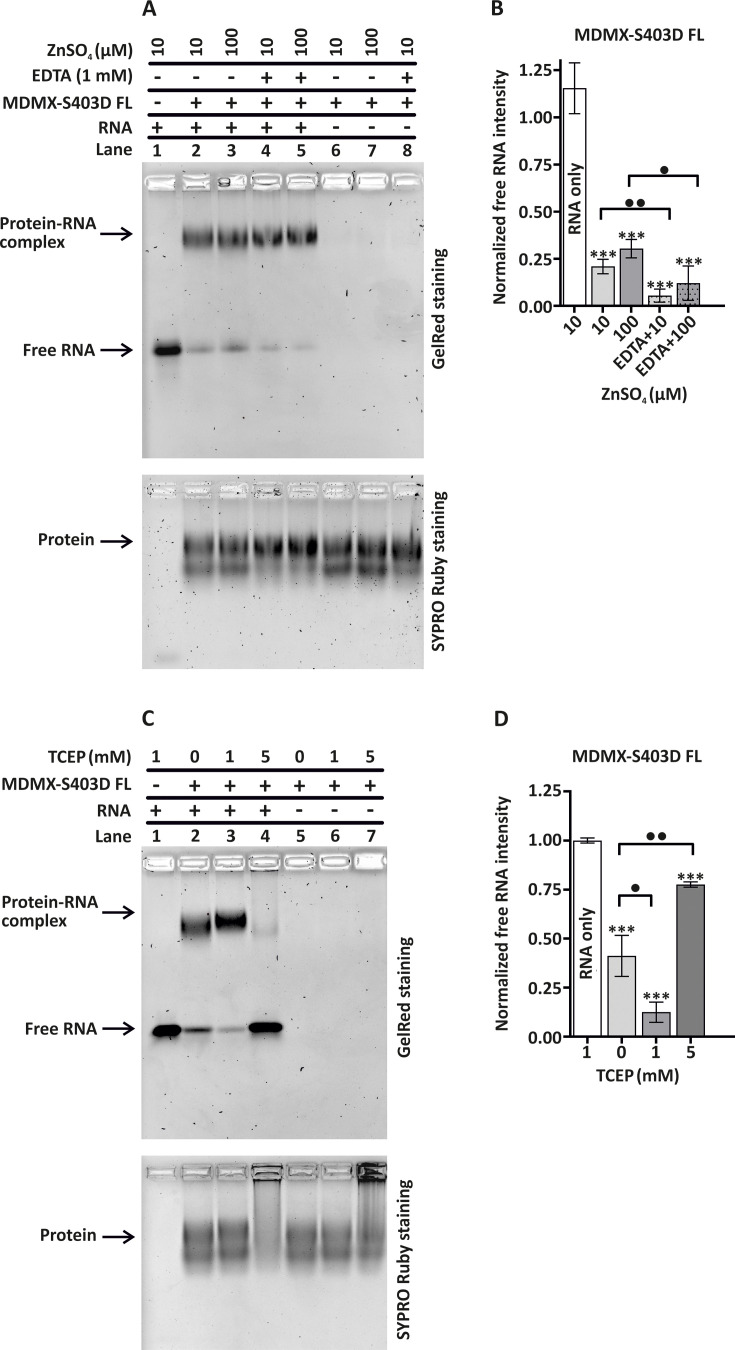
Modulation of *TP53* mRNA–MDMX-S403D FL interaction by zinc, EDTA, and reducing conditions. (**A**) EMSA analysis of *TP53* mRNA binding to MDMX-S403D FL construct in the presence of Zn²^+^ and EDTA. Proteins were incubated with *TP53* mRNA (lanes 2–5) or without RNA (lanes 6–8) in binding buffer (150 mM NaCl, 50 mM Tris, 1 mM TCEP, pH 7.5) and either 10  µM or 100  µM ZnSO₄, in the presence of EDTA (dotted columns) or absence of EDTA (empty columns). Free RNA (lane 1) served as a control. (**B, D**) Quantification of free RNA band intensity from gels shown in (**A**) and (**C**), respectively, normalized to their corresponding RNA-only control. Data represent mean ± SEM (*n* = 3 biological replicates; ***P<0.001). Additional statistical comparisons are shown in the figure and alternatively indicated as •*P*<0.05 and ••*P*<0.01. (**C**) EMSA showing the effect of the reducing agent TCEP on *TP53* mRNA to MDMX-S403D FL construct. Proteins were incubated with *TP53* mRNA (lanes 2–4) or without RNA (lanes 5–7) in binding buffer containing 0, 5, or 10  mM TCEP. Free RNA (lane 1) served as a control. In panels (**A**) and (**C**), RNA–protein complexes were visualized by GelRed staining (upper panels), and total protein was detected using SYPRO Ruby staining of the same gels (lower panels). The RNA:protein molar ratio was 1:50 in all reactions.

We hypothesized that MDMX binds RNA via its RING domain, with other domains potentially masking the interaction interface. To characterize the structural features within the RING domain that are critical for RNA interaction, we examined the role of zinc co-ordination and disulfide bond integrity in MDMX-S403D FL binding. The RING domain of MDMX consists of a C2H2C4 structure with two cysteines (C1 and C2), two histidines (H3 and H4), and four cysteines (C5–C8), which co-ordinate two Zn²^+^ ions essential for stabilizing its folded state [21,22]. Moreover, MDMX contains an additional Zn-finger domain spanning residues 290–322. To investigate the role of zinc ions and their chelation by EDTA in RNA binding, we performed EMSA using the MDMX-S403D FL protein (Figure 4A, quantified in 4B) and the C-terminal variant MDMX-S403D (322–490) (Supplementary Figure S4A), quantified in S4B) in the presence of zinc concentrations promoting proper protein folding (10 µM Zn²^+^), an excess of free Zn²^+^ (100 µM), and 1 mM EDTA combined with varying zinc concentrations. For MDMX-S403D FL, excess zinc ions revealed a decrease in RNA binding seen as free RNA increase (Figure 4A, upper panel, lane 3). In contrast, the addition of EDTA enhanced RNA binding significantly, suggesting that zinc chelation by EDTA facilitates interaction with RNA (Figure 4A, upper panel, lane 4 and 5, quantified in Figure 4B). Similar trends in RNA changes were observed for the MDMX-S403D (322–490) variant (Supplementary Figure S4A and S4B), and upon EDTA addition, this was accompanied by disappearance of the smear that had indicated RNA–protein complex formation (Supplementary Figure S4A, upper panel, lanes 4 and 5). It has been proposed that EDTA may induce unfolding and potential aggregation of RING finger domains [23]. To examine this effect, we used SYPRO Ruby staining to evaluate protein mobility in agarose gels, in both RNA-bound and RNA-free forms. MDMX-S403D FL formed two bands even in the absence of RNA (Figure 4A, lower panel, lanes 6–8). No significant change in mobility was observed upon Zn²^+^ treatment (lanes 3 and 7), indicating no major structural changes occurred. However, in the presence of 1 mM EDTA, we observed reduction of the lower protein band in both RNA-bound (lanes 4 and 5) and RNA-free forms (lane 8), suggesting a structural alteration of the protein. By overlaying GelRed and SYPRO Ruby staining, we identified the upper band as the RNA-binding form (Supplementary Figure S4E), consistent with EDTA-induced structural rearrangement that promotes RNA interaction. The C-terminal variant MDMX-S403D (322–490) showed no mobility changes in response to zinc ions (Supplementary Figure S4A, lower panel, lanes 2, 3, 6, and 7). However, the addition of 1 mM EDTA induced accumulation of the protein in the gel wells accompanied by the disappearance of the smear, suggesting protein aggregation (Supplementary Figure S4A, lower panel, lanes 4, 5, and 8). Together, these results indicate that zinc ions interfere with RNA binding, while EDTA enhances binding, presumably by chelating zinc and unmasking the interface. However, EDTA probably weakens the structural stability of the protein, particularly of the C-terminal domain. These findings highlight the delicate balance between structural integrity and accessibility in the regulation of RNA binding by the MDMX RING domain.

The MDMX protein contains cysteine residues, primarily located in the zinc finger and RING domains, which can form disulfide bonds involved in protein folding and oligomerization. However, under reducing conditions prevalent in the cellular environment, disulfide bonds become unstable. To investigate the role of disulfide bonds in RNA binding, we used increasing concentration of TCEP, a potent reducing agent, and analyzed RNA-binding capacity using EMSA (Figure 4C and quantified in 4D). RNA–protein complex formation was observed under native conditions without TCEP (lane 2) and increased upon treatment with 1 mM TCEP (lane 3). However, at 5 mM TCEP, complex formation by MDMX-S403D FL was disrupted, indicated by the reappearance of free RNA (lane 4). SYPRO Ruby confirmed that 1 mM TCEP did not alter the migration of MDMX-S403D FL (Figure 4C, lower panel, lane 3), whereas 5 mM TCEP probably caused aggregation, with protein retained in the well (lane 4). A similar aggregation was observed for the RNA-free protein at 5 mM TCEP (lane 7). For MDMX-S403D (322–490) (Supplementary Figure S4C, and quantified in S4D), we observe a decrease in free RNA upon 1 mM and 5 mM TCEP addition. However, the SYPRO staining reveals (Supplementary Figure S4C, lower panel) that 1 mM TCEP enhanced gel penetration of both RNA-bound and RNA-free forms (lanes 3 and 6), indicating disulfide bond disruption. In contrast, 5 mM TCEP again led to protein retention in the well, suggesting aggregation (lanes 4 and 7). These results suggest that excessive disulfide bond formation can partially obstruct RNA binding, whereas their complete absence disrupts protein integrity. In summary, disulfide bonds are essential for both the RNA-binding ability and the structural stability of MDMX.

**Figure 5:**
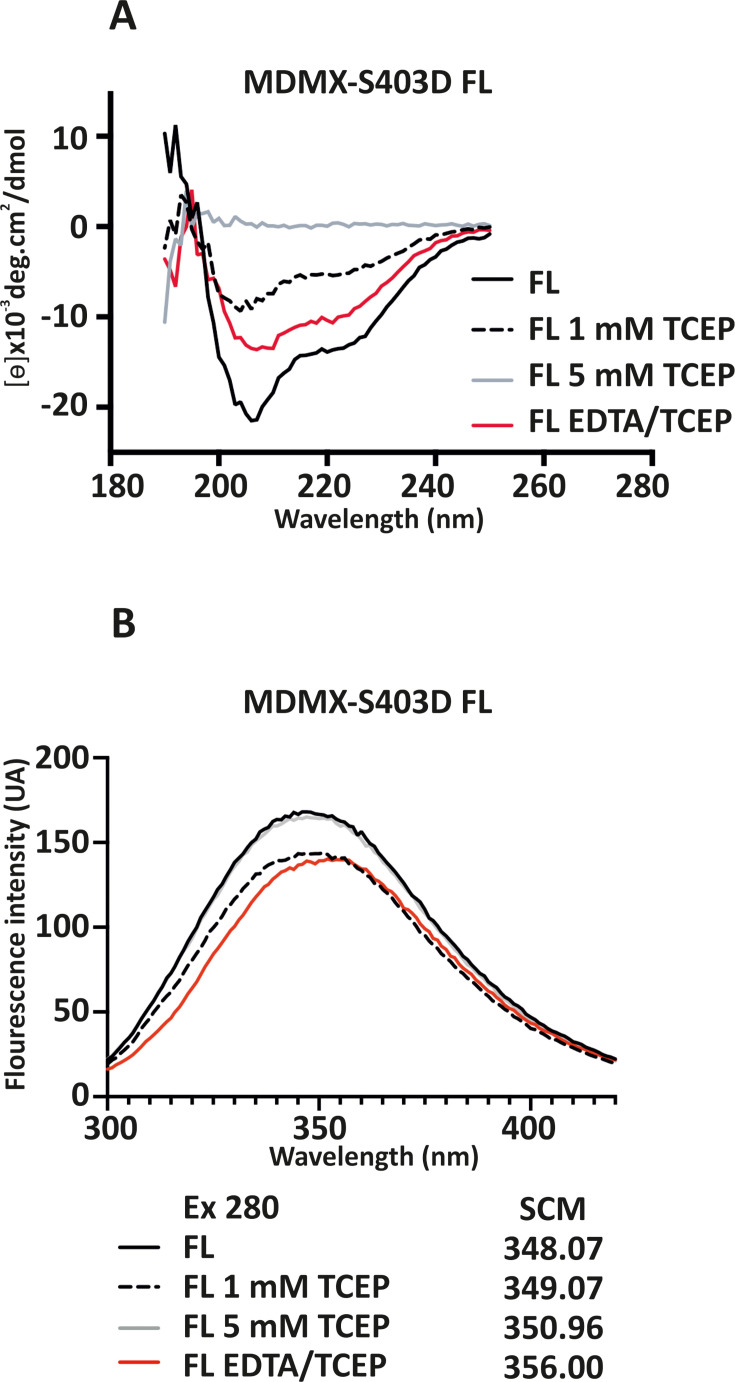
Effects of TCEP and EDTA on conformation of MDMX-S403D FL. (**A**) CD spectroscopy of MDMX-S403D FL measured in 10  mM sodium phosphate buffer (pH 7.5, without NaCl) across the wavelength range 180–280  nm. (**B**) Intrinsic fluorescence emission spectra of MDMX-S403D FL measured in RNA-binding buffer (150  mM NaCl, 20  mM Tris-HCl, pH 7.5) upon excitation at 280  nm. In both (**A**) and (**B**), measurements were performed under four conditions: untreated (black line), with 1  mM TCEP (black dashed line), with 5  mM TCEP (gray line), and with a combination of 1  mM EDTA and 1  mM TCEP (red line).

To assess how EDTA and TCEP modulate MDMX structure and RNA-binding ability, we analyzed their effects on protein conformation using CD spectroscopy and intrinsic fluorescence. The far-UV CD measurements of secondary structure (180–280 nm) were performed for MDMX-S403D FL (Figure 5A) and C-terminal MDMX-S403D (322–490) (Supplementary Figure S5A) in phosphate buffer without NaCl to avoid spectral interference. The CD spectrum of untreated MDMX-S403D FL displayed two negative peaks at ~210  nm and ~225  nm, characteristic of folded secondary structure (Figure 5A, black line). Upon addition of 1 mM TCEP, partial denaturation was observed (black dashed line), whereas 5 mM TCEP caused complete denaturation (gray line). Notably, the addition of 1  mM EDTA in the presence of 1  mM TCEP (red line), as used in the RNA binding assay (Figure 4A), preserved the CD profile, suggesting that EDTA protects the secondary structure under reducing conditions. This effect may be partially due to oxidation of TCEP by EDTA. To examine the role of EDTA independently, we analyzed its effect in the absence of TCEP, which resulted in a partial loss of structural integrity (Supplementary Figure S5C). The CD spectrum of the C-terminal MDMX-S403D (322–490) construct did not display a well-defined peak (Supplementary Figure S5A), likely due to the presence of an intrinsically disordered region upstream of the structured RING domain. However, a measurable CD signal confirmed that the protein was folded under control conditions (black line). TCEP decreased the CD signal (black dashed line), indicating denaturation. In contrast to the FL protein, EDTA did not restore the secondary structure in this truncated variant (red line).

To complement CD data, we analyzed intrinsic protein fluorescence emission profiles for both constructs using the same buffer conditions as the RNA-binding assays. This method detects conformational shifts by monitoring changes in the exposure of fluorescent amino acid residues (Trp, Tyr, Phe). For both MDMX-S403D FL (Figure 5B) and C-terminal MDMX-S403D (322–490) (Supplementary Figure S5B), minor spectral shifts were observed upon treatment with 1  mM and 5  mM TCEP (black dashed and gray lines). A more pronounced shift in the spectral center of mass (SCM) was observed when EDTA was combined with TCEP (red line), suggesting that the protein conformation is more affected under these conditions.

To evaluate the functional consequences of these conformational changes, EMSA was performed using buffer conditions used in fluorescence experiments (Supplementary Figure S5D and quantified in 5SE). Consistent with prior observations (Figure 4A and C), the MDMX-S403D FL protein formed RNA–protein complex in the absence of TCEP and EDTA (Supplementary Figure S5D, lane 2), and complex formation increased in the presence of 1  mM TCEP (Supplementary Figure S5D, lane 3). In contrast, 1  mM EDTA alone did not enhance complex formation (Supplementary Figure S5D, lane 4). The strongest binding was observed when both 1  mM TCEP and 1  mM EDTA were present during binding (Supplementary Figure S5D, lane 5). SYPRO Ruby staining (Supplementary Figure S5D, lower panel) showed that EDTA alone increased the intensity of the upper protein band (lanes 4 and 5), previously identified as the RNA-binding form (Supplementary Figure S5E), even in the absence of TCEP (lane 4). Altogether, these results demonstrate that the combined presence of 1 mM TCEP and 1 mM EDTA reduces structural rigidity in MDMX-S403D FL and facilitates its interaction with RNA.

## Discussion

MDMX interaction with p53 and MDM2 is well studied, but its binding to *TP53* mRNA and how this affects its other interactions remains less understood. After DNA damage, both MDMX and MDM2 bind to the IRES region of *TP53* mRNA to regulate its translation. MDMX first binds and folds *TP53* mRNA, enabling MDM2 to enhance translation. This process has been elucidated using various mRNA mutants [8]. Recently, ATM kinase, a key player in the DNA damage response, was identified as another *TP53* mRNA binding partner. Notably, ATM competes with MDMX for *TP53* mRNA, although the mechanisms regulating their competition remain unclear [9]. In this study, we characterized the MDMX-RNA binding region and identified its key structural features. Using full-length and truncated constructs of both WT and the phosphomimetic mutant MDMX-S403D (Figure 1A), we confirmed that the S403D mutation enhances binding affinity. We showed the protein variant without the RING domain does not bind efficiently to RNA, and the C-terminal variant (322-490) eliminates the free RNA efficiently, with RNA complex appearing as a smear. Although protein aggregation cannot be entirely ruled out, the data support a primary role for the RING domain in RNA binding. The FL variant and N-terminally truncated constructs may be sterically masked by structural organization of protein domains. HDX-MS (Figure 2A) did not reveal specific RNA-contacting residues but instead showed changes throughout the protein, including a slight opening of the N-terminal domain. This rearrangement may impair RNA–protein binding in a manner similar to how the *TP53* mRNA binding to MDM2 prevents its interaction with the p53 protein [24]. We speculate that the absence of a clearly defined RNA-binding interface is due to a combination of several factors: the intrinsic flexibility of MDMX [12], the low accessibility of the RING domain for deuteration (as indicated by the low deuteration rate of the RING domain, supported by EMSA results showing the RING domain is masked for RNA interaction in MDMX-S403D FL), and the lack of a well-defined binding site, likely due to the dispersed positively charged residues within the RING domain that mediate electrostatic RNA interaction. These interpretations are further supported by EMSA, showing that the RING domain appears structurally masked in the MDMX-S403D FL variant. We confirmed that binding of the MDMX C-terminal domain to *TP53* mRNA is primarily electrostatic as the complex is released by high salt concentration. Notably, surface interactions, even in the absence of specific contacts, can still enable high-affinity binding [25]. In contrast, the complex formed by MDMX-S403D FL and *TP53* mRNA remained stable at elevated salt concentrations (Figure 2C), further supporting the involvement of additional domains in RNA binding. Similarly, the FL construct maintained complex stability under varying pH conditions (Figure 3A). These findings underscore the importance of studying the RING domain function within the context of its biologically relevant form. To further validate and expand our findings, future studies should employ structural approaches such as cryo-electron microscopy, X-ray crystallography, NMR spectroscopy, and cross-linking mass spectrometry to resolve RNA–protein binding interfaces and conformational dynamics in more physiological contexts.

Given that the RING domain is structurally stabilized by co-ordinated Zn²^+^ ions, we next examined how zinc availability influences RNA binding. An excess of Zn²^+^, similar to Mg^2+^ ions [8], likely interferes with binding by promoting RNA folding [26]. In contrast, the addition of EDTA enhanced binding by chelating zinc. However, this chelation may also unfold zinc-stabilized structures, including the RING domain itself [22]. The structural changes observed by CD spectroscopy and intrinsic fluorescence suggest that EDTA exerts a protective effect on the secondary structure of the MDMX-S403D FL protein under reducing conditions (1 mM TCEP), while simultaneously promoting structural opening, as indicated by an increase in intrinsic fluorescence signal. A less constrained protein conformation likely contributes to enhanced RNA binding by increasing accessibility of the interaction interface. However, the structural changes induced by EDTA in the absence of TCEP were insufficient to enhance RNA binding. Considering that *TP53* mRNA interacts with multiple proteins and is tightly regulated [27], MDMX likely competes with other factors, such as ATM, which has also been reported to modulate MDMX activity in the context of RNA binding [9]. Cellular conditions (pH, ionic strength, reducing environment) can fluctuate during cancer progression or cellular stress, potentially influencing MDMX’s choice of binding partner. Ongoing research has largely focused on the development of MDMX inhibitors. To date, these compounds primarily block the interaction between p53 and the N-terminal p53-binding pocket of MDMX [28,29]. Several inhibitors are in clinical evaluation, including ALRN-6924, the first stapled peptide dual inhibitor of MDM2/MDMX, which has shown encouraging activity in early phase trials [30,31]. In parallel, small molecules such as the MMRi series have been reported to target the RING domain of the MDM2-MDMX E3 ligase complex, thereby disrupting ubiquitin ligase activity and activating the p53 pathway [32,33]. Despite these advances, the broader consequences of such inhibitors remain insufficiently understood. In particular, their potential effects on MDMX functions beyond p53 and MDM2 regulation, such as RNA binding, warrant further investigation, as these may exert wider cellular impacts.

MDMX plays its role of RNA chaperone for *TP53* mRNA, with electrostatic forces probably playing a key role in this process. RNA chaperones are often intrinsically disordered proteins that act as polyanions, facilitating proper folding of nucleic acids [34]. These proteins typically form transient interactions with RNA and are then released from the folded RNA [35]. In this context, the C-terminal domain of MDMX appears to enable the transient binding as it shows sensitivity to changing conditions such as pH and ionic strength. Other domains of MDMX may play complementary roles here by stabilizing the RNA–protein complex and preventing premature release, thus adding an additional layer of regulation to the proposed chaperone activity of MDMX.

Although our study provides detailed mechanistic insights into the structural basis of MDMX–RNA interaction, it relies on *in vitro* experiments using a synthetic fragment of *TP53* mRNA. While this reductionist approach enabled us to characterize the underlying mechanism, establishing physiological relevance will require studies in cellular systems. Previous studies have demonstrated that *TP53* mRNA can modulate MDM2/MDMX activity in cells and that these RNA–protein complexes are detectable by proximity ligation assays (PLA) and co-immunoprecipitation, supporting the feasibility of validation for this axis in cells [18,24]. Similarly, ATM kinase has been shown to interact with the *TP53* mRNA, further highlighting the RNA-mediated regulation within the MDM2/MDMX network in cells [7,9]. In this context, it will be to test whether the conditions that enhanced binding *in vitro* (phosphomimetic S403D mutation, altered ionic strength, Zn²^+^ availability, and redox state) also modulate MDMX–*TP53* mRNA interactions in cells. This could be addressed by expressing MDMX variants (WT/S403D), manipulating Zn²^+^ levels through chelation or supplementation, and applying controlled redox perturbations, with RIP/eCLIP and RNA-PLA providing complementary readouts. Implementing these strategies may clarify the physiological relevance of MDMX conformational flexibility for *TP53* mRNA binding and its role in regulating the p53 pathway *in vivo*.

Importantly, these interactions do not occur in isolation. In cells, MDMX forms a complex with MDM2, and this partnership may influence RNA-binding affinity and specificity, either by helping the complex bind to *TP53* mRNA or by inducing structural changes that affect the binding interface [36]. Therefore, future research should also consider the dynamic stoichiometry and potential co-operative effects of the MDMX–MDM2 complex when studying *TP53* mRNA interactions *in vivo*.

Altogether, these findings uncover features of the MDMX–RNA interaction, providing mechanistic insight into how MDMX may regulate *TP53* mRNA translation. This expanded understanding of MDMX function highlights its potential role in RNA-mediated gene regulation and underscores the importance of considering its noncanonical activities in cancer biology.

## Supplementary material

online supplementary figure 1

online supplementary figure 2

online supplementary figure 3

online supplementary figure 4

online supplementary figure 5

online supplementary material 1

## Data Availability

The data supporting the conclusions of this article are available from the corresponding author on reasonable request.

## Abbreviations

ATM: ataxia-telangiectasia mutated
CD: circular dichroism
EMSA: electrophoretic mobility shift assay
FL: full-length
HDX-MS: hydrogen-deuterium exchange mass spectrometry
IRES: internal ribosome entry site
MDM2: mouse double minute 2 homolog
MDMX: mouse double minute X homolog
NBS1: Nijmegen breakage syndrome 1
SDS-PAGE: sodium dodecyl sulfate-polyacrylamide gel electrophoresis
WT: wildtype
