# Supplementary figures and images for: Reduced structural rigidity of MDMX protein enhances binding to TP53 mRNA
Source: Biosci Rep. 2025 Nov 25;45(11):683–96. doi: 10.1042/BSR20253646 (PMC12751038; doi:10.1042/BSR20253646)

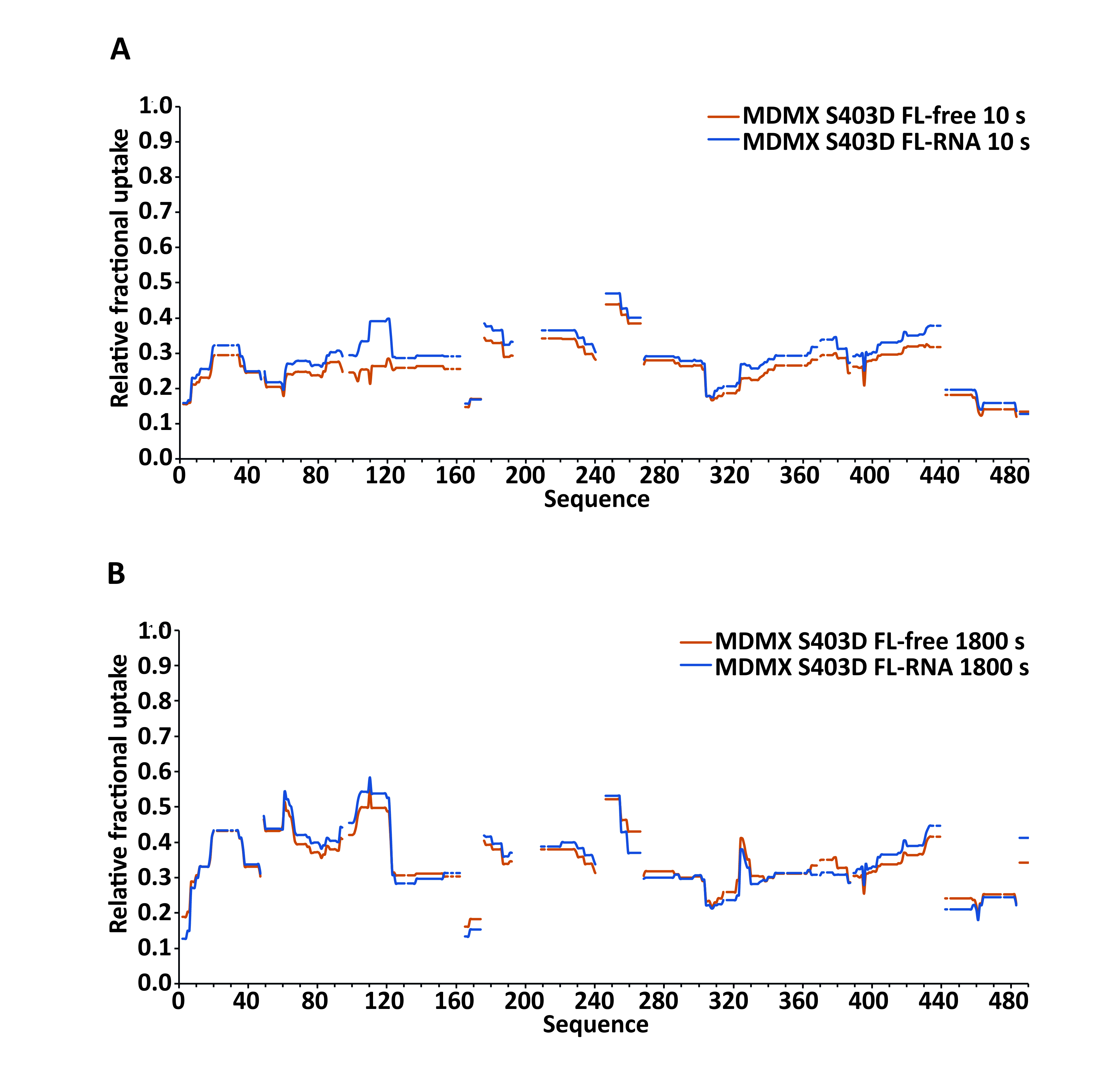

Supplement: online supplementary figure 1 [file bsr-45-11-BSR20253646-s001.tif]

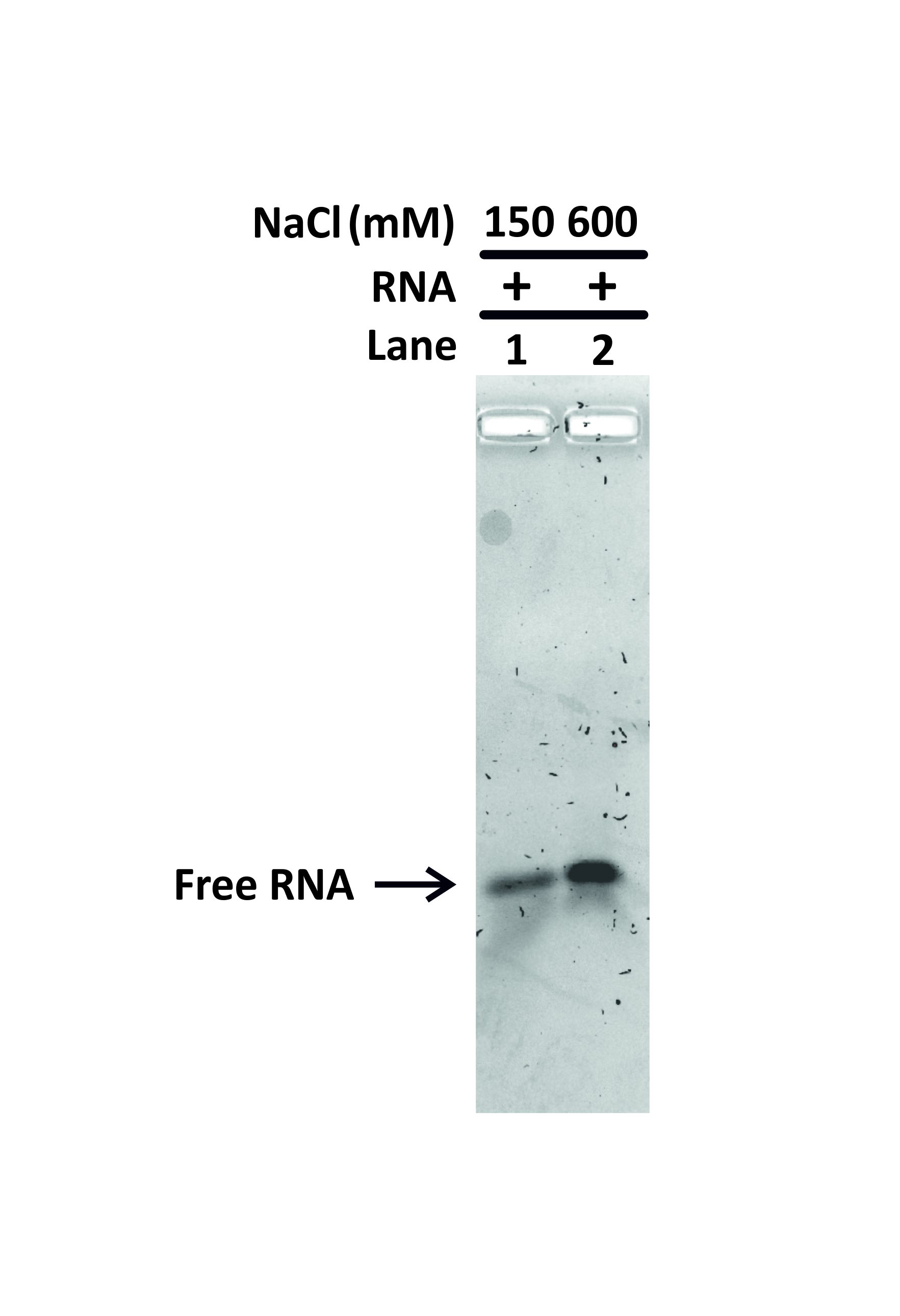

Supplement: online supplementary figure 2 [file bsr-45-11-BSR20253646-s002.tif]

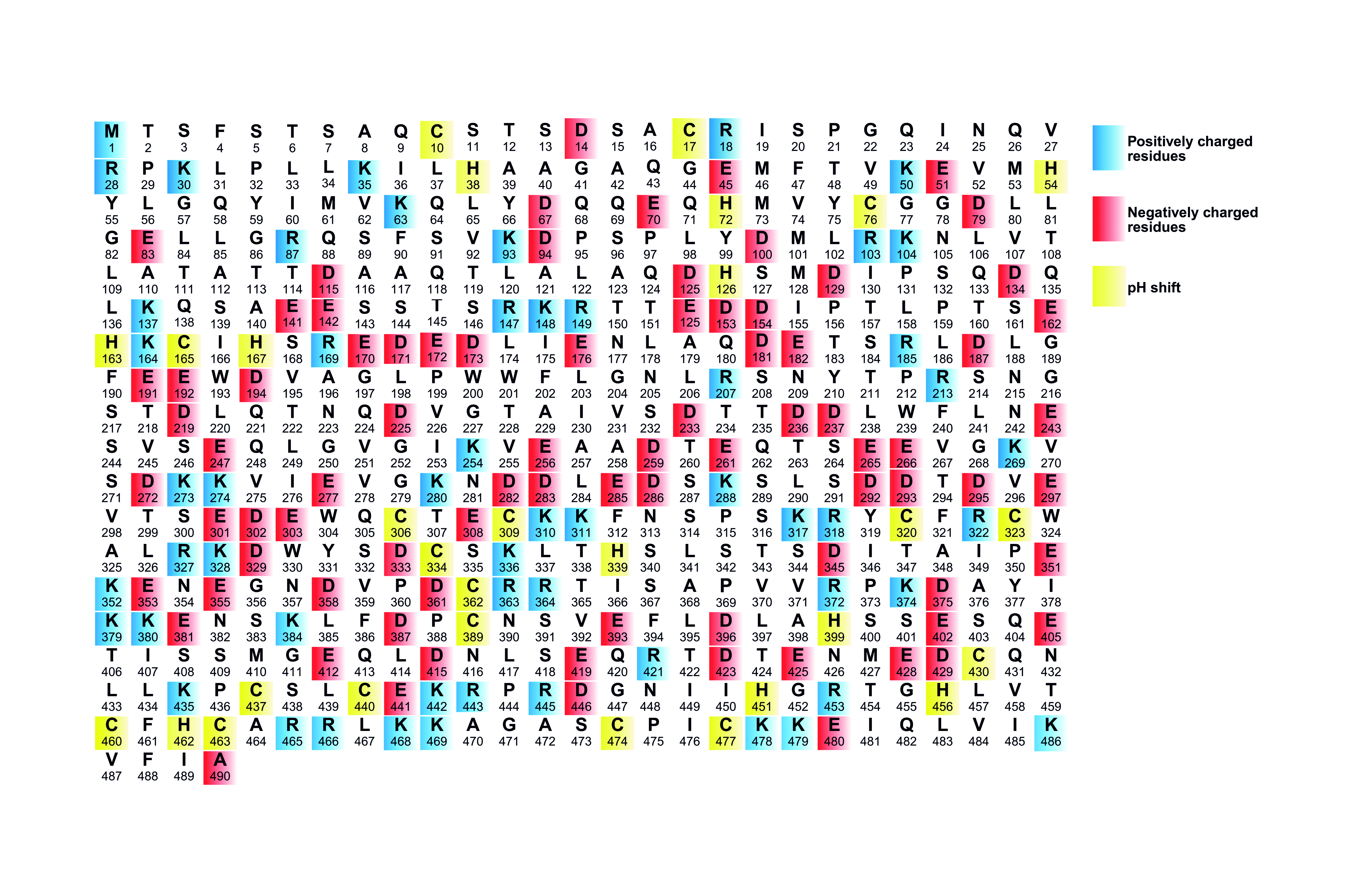

Supplement: online supplementary figure 3 [file bsr-45-11-BSR20253646-s003.tif]
