# Supplementary material for: Reduced structural rigidity of MDMX protein enhances binding to TP53 mRNA
Source: Biosci Rep. 2025 Nov 25;45(11):683–96. doi: 10.1042/BSR20253646 (PMC12751038; doi:10.1042/BSR20253646)
Supplement: online supplementary material 1 [file bsr-45-11-BSR20253646-s006.pdf]

Raw Data Supplement + Replicates

Reduced structural rigidity of MDMX protein enhances binding to *TP53* mRNA

Raw data for Figure 1B

EMSA = Interaction between MDMX-S403D FL protein construct and *TP53* mRNA (GelRed staining)

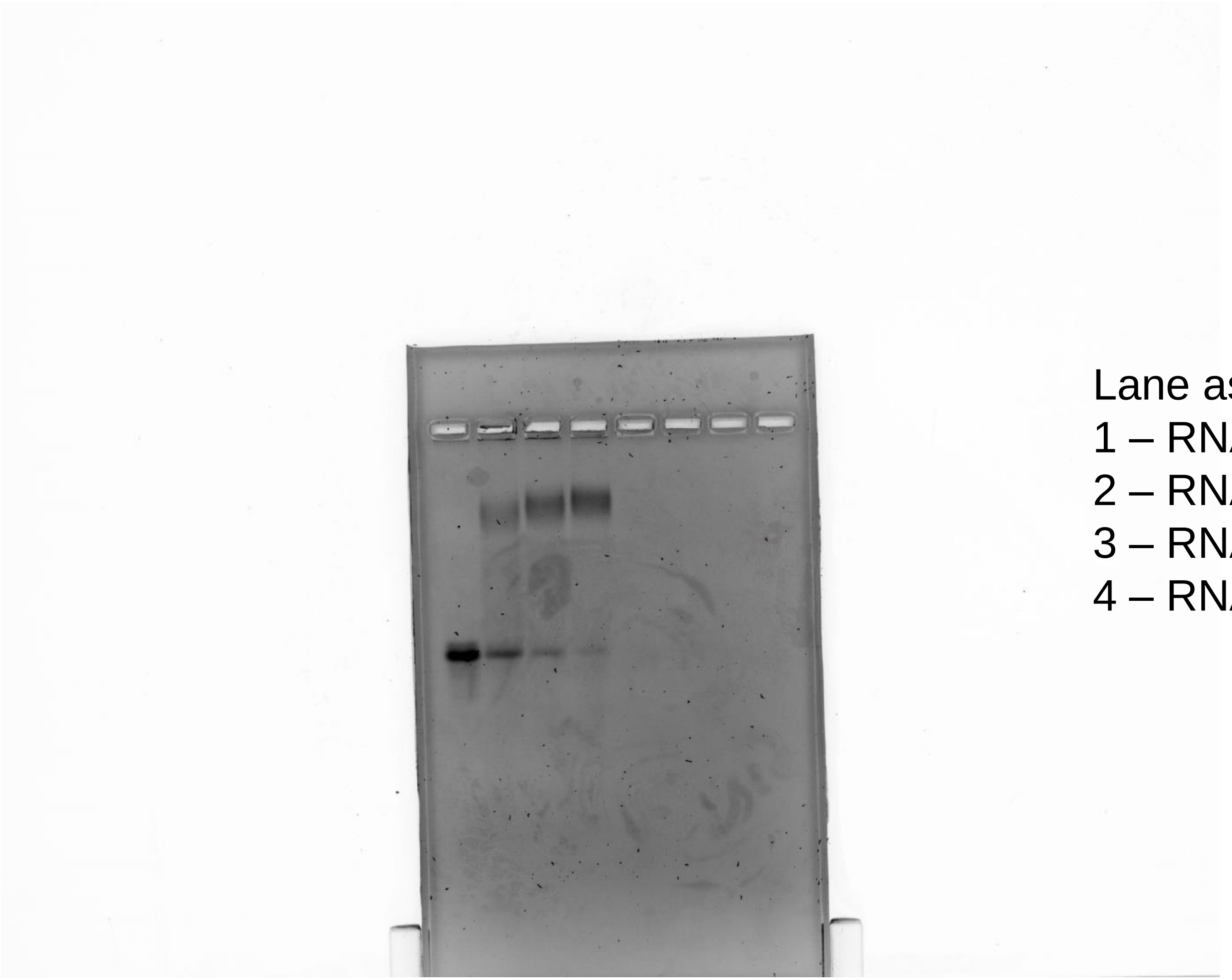

Lane assignment:
1 – RNA (ratio 1:0)
2 – RNA + MDMX-S403D FL (ratio 1:25)
3 – RNA + MDMX-S403D FL (ratio 1:50)
4 – RNA + MDMX-S403D FL (ratio 1:100)

Replicates for Figure 1B

EMSA = Interaction between MDMX-S403D FL protein construct and *TP53* mRNA (GelRed staining)

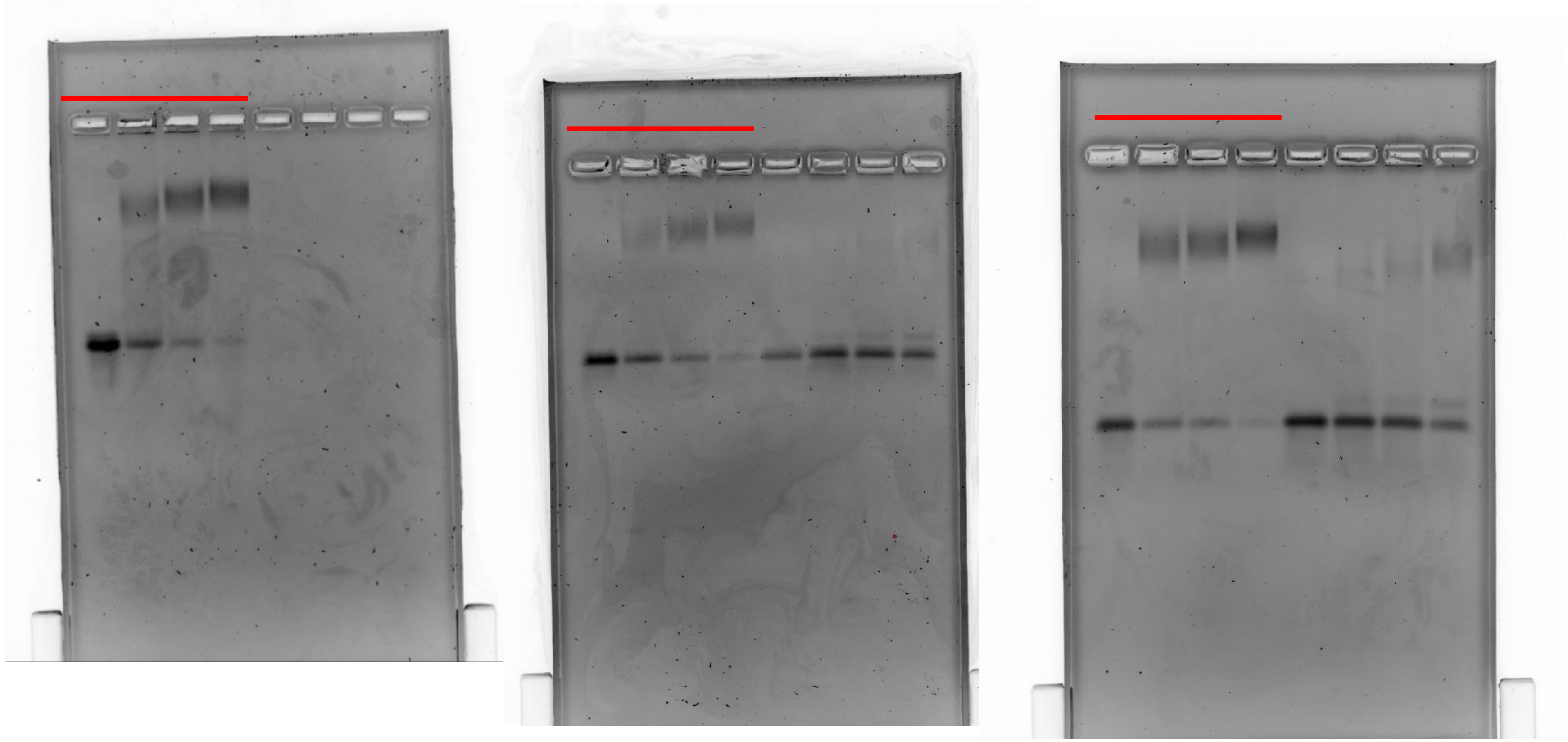

Lane assignment:
1 – RNA (ratio 1:0)
2 – RNA + MDMX-S403D FL (ratio 1:25)
3 – RNA + MDMX-S403D FL (ratio 1:50)
4 – RNA + MDMX-S403D FL (ratio 1:100)

Raw data for Figure 1C

EMSA = Interaction between MDMX-S403D (128-490) protein construct and *TP53* mRNA (GelRed staining)

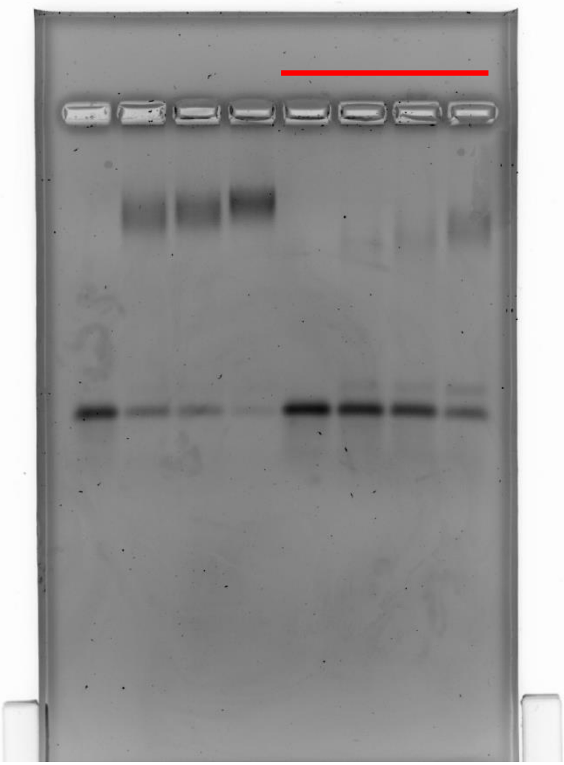

Lane assignment (gel lanes 1-4):
1 – RNA (ratio 1:0)
2 – RNA + MDMX-S403D (128-490) (ratio 1:25)
3 – RNA + MDMX-S403D (128-490) (ratio 1:50)
4 – RNA + MDMX-S403D (128-490) (ratio 1:100)

Triplicates for Figure 1C

EMSA = Interaction between MDMX-S403D (128-490) protein construct and *TP53* mRNA (GelRed staining)

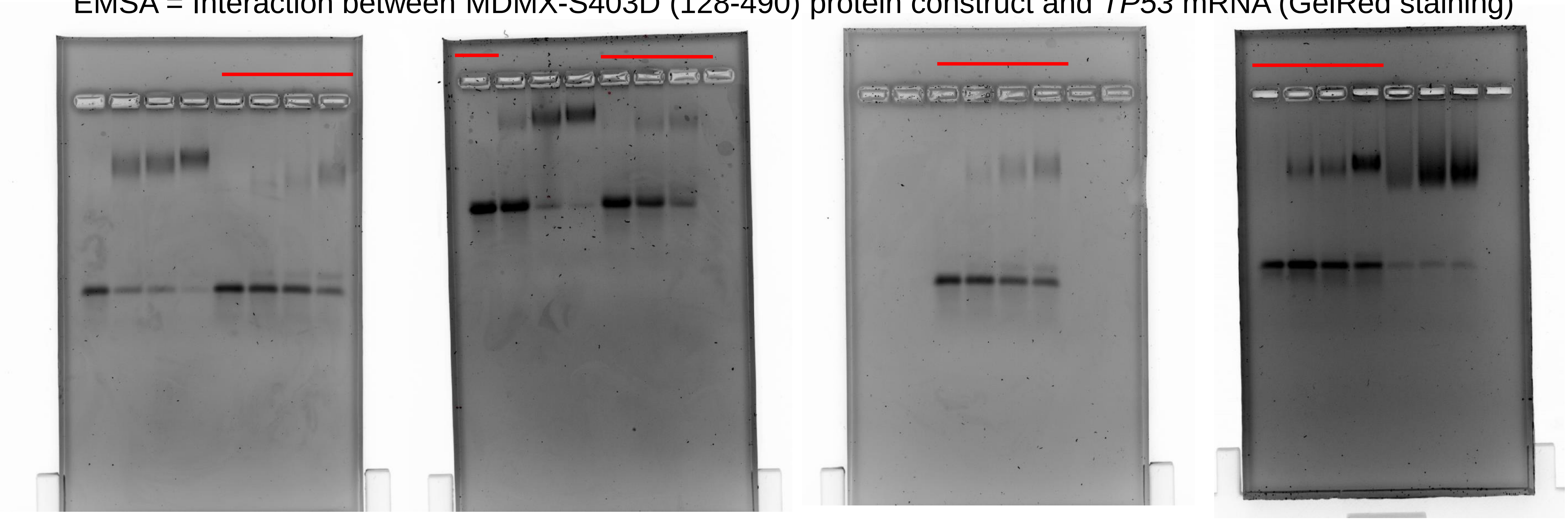

Lane assignment (gel lanes 1-4):
1 – RNA (ratio 1:0)
2 – RNA + MDMX-S403D (128-490) (ratio 1:25)
3 – RNA + MDMX-S403D (128-490) (ratio 1:50)
4 – RNA + MDMX-S403D (128-490) (ratio 1:100)

Raw data for Figure 1D

EMSA = Interaction between MDMX-S403D (1-436) protein construct and *TP53* mRNA (GelRed staining)

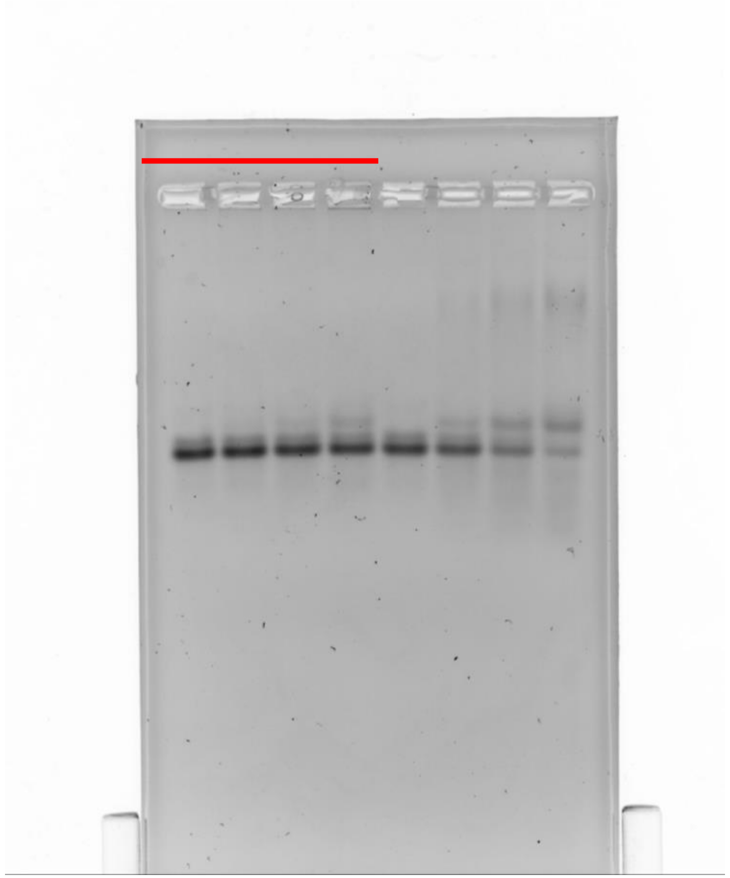

Lane assignment (gel lanes 1-4):
1 – RNA (ratio 1:0)
2 – RNA + MDMX-S404D (1-436) (ratio 1:25)
3 – RNA + MDMX-S404D (1-436) (ratio 1:50)
4 – RNA + MDMX-S404D (1-436) (ratio 1:100)

Replicates for Figure 1D

EMSA = Interaction between MDMX-S403D (1-436) protein construct and *TP53* mRNA (GelRed staining)

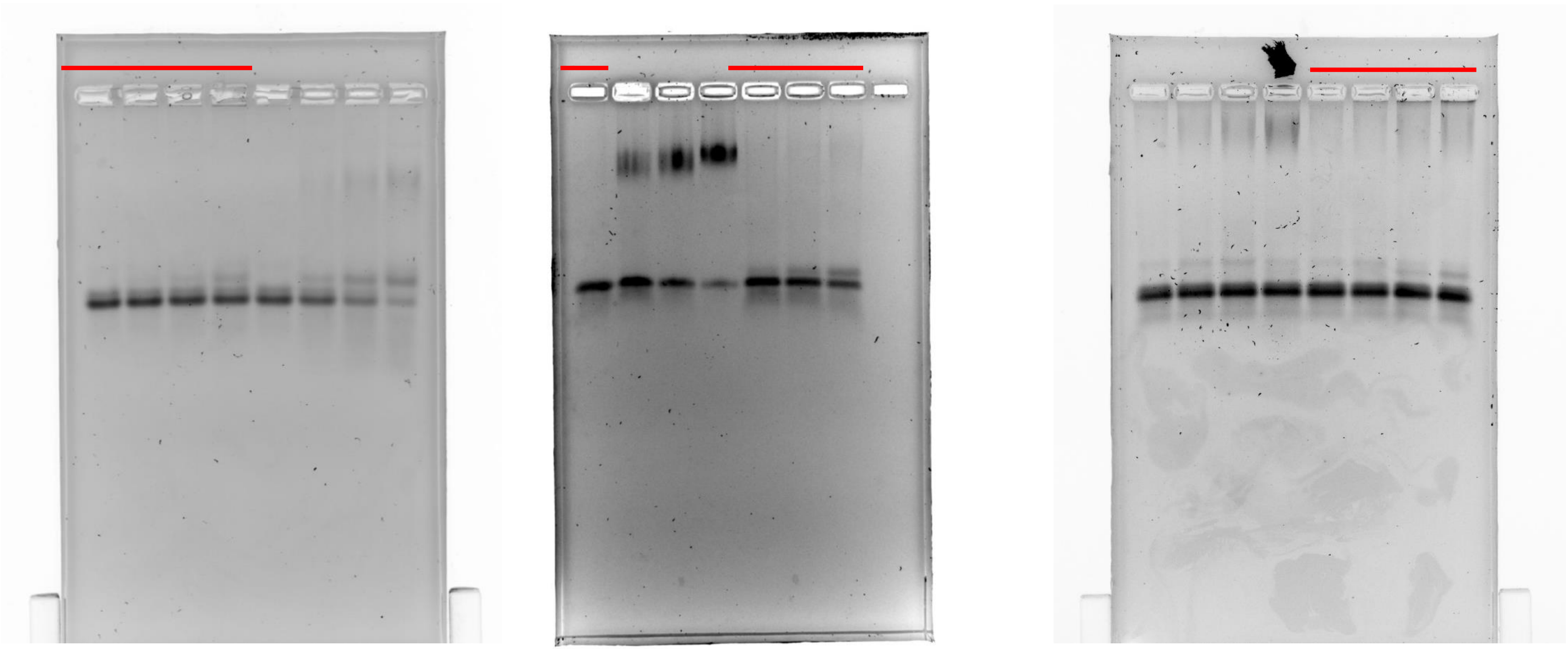

Lane assignment (gel lanes 1-4):
1 – RNA (ratio 1:0)
2 – RNA + MDMX-S404D (1-436) (ratio 1:25)
3 – RNA + MDMX-S404D (1-436) (ratio 1:50)
4 – RNA + MDMX-S404D (1-436) (ratio 1:100)

Raw data for Figure 1E

EMSA = Interaction between MDMX-S403D (322-490) protein construct and *TP53* mRNA (GelRed staining)

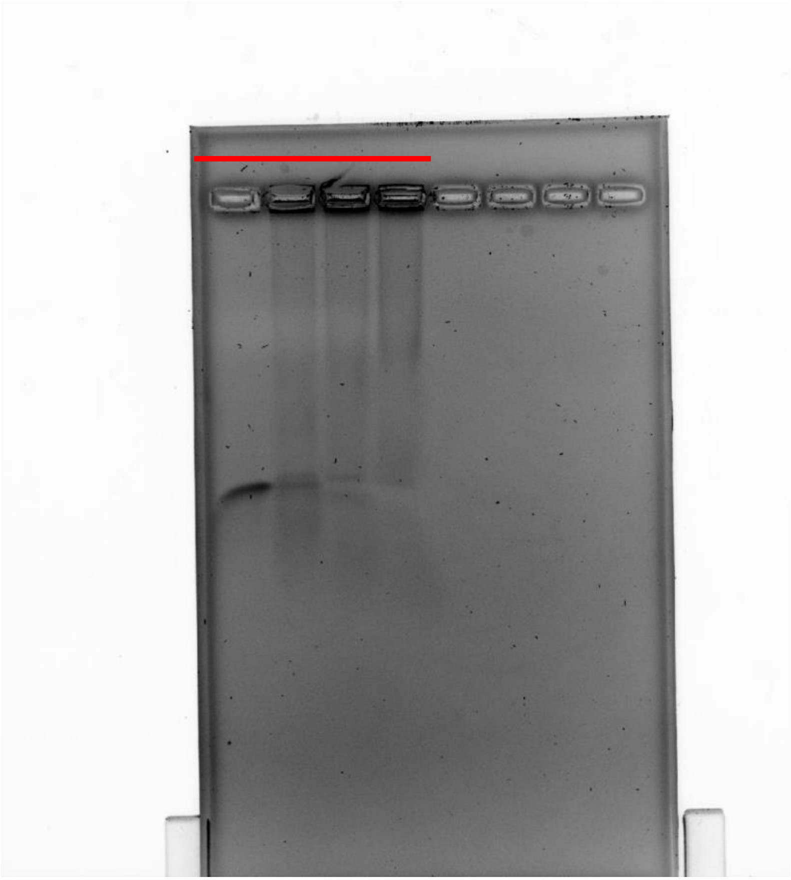

Lane assignment:
1 – RNA (ratio 1:0)
2 – RNA + MDMX-S403D (322-490) (ratio 1:25)
3 – RNA + MDMX-S403D (322-490) (ratio 1:50)
4 – RNA + MDMX-S403D (322-490) (ratio 1:100)

Replicates for Figure 1E

EMSA = Interaction between MDMX-S403D (322-490) protein construct and *TP53* mRNA (GelRed staining)

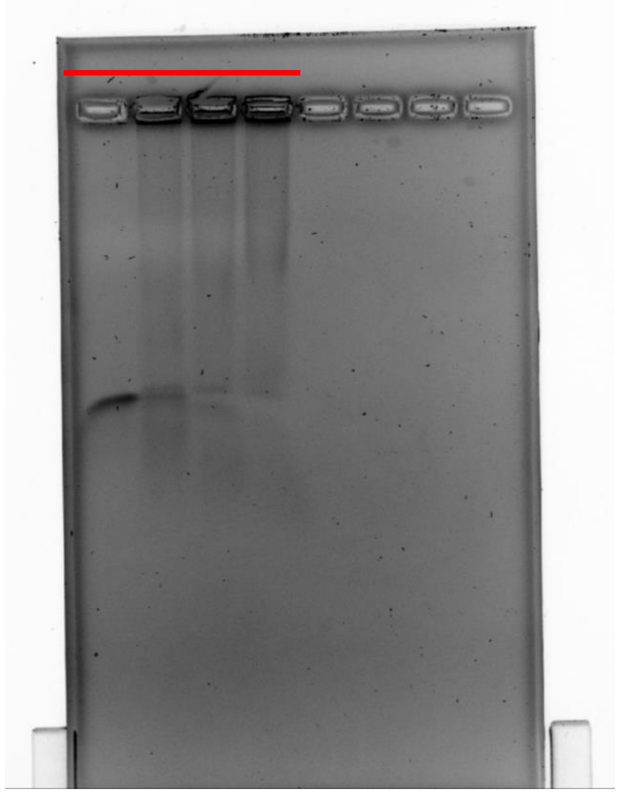

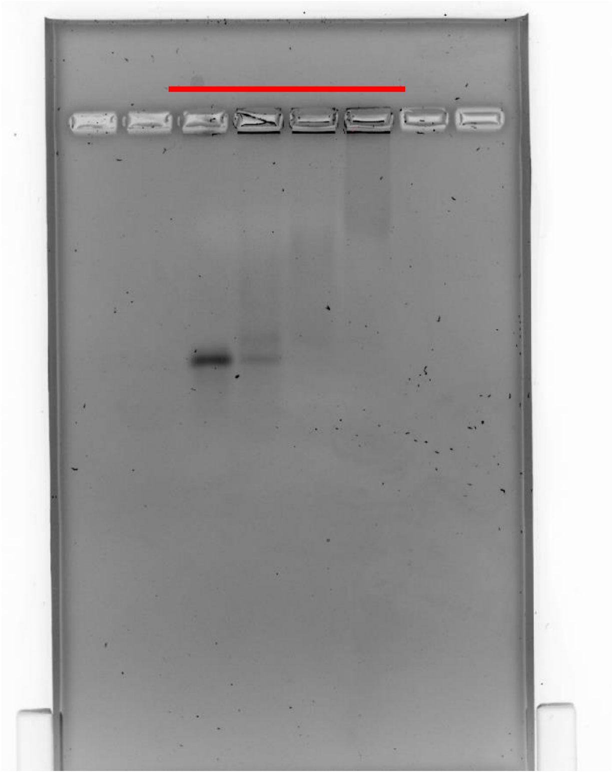

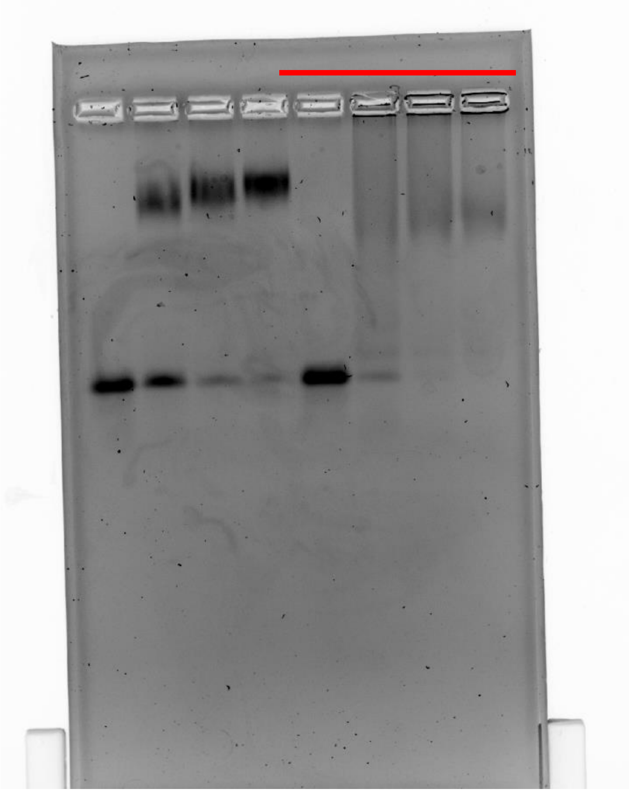

Lane assignment:
1 – RNA (ratio 1:0)
2 – RNA + MDMX-S403D (322-490) (ratio 1:25)
3 – RNA + MDMX-S403D (322-490) (ratio 1:50)
4 – RNA + MDMX-S403D (322-490) (ratio 1:100)

Raw data for Figure 1F

EMSA = Interaction between MDMX WT FL protein construct and *TP53* mRNA (GelRed staining)

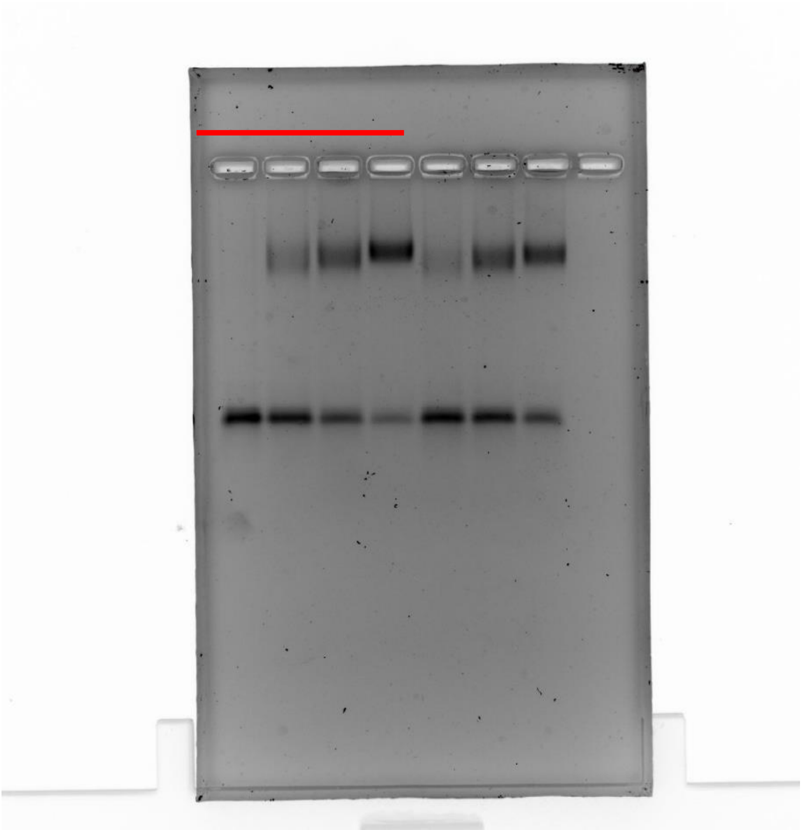

Lane assignment (gel lanes 1-4):
1 – RNA (ratio 1:0)
2 – RNA + MDMX WT FL (ratio 1:25)
3 – RNA + MDMX WT FL (ratio 1:50)
4 – RNA + MDMX WT FL (ratio 1:100)

Replicates for Figure 1F

EMSA = Interaction between MDMX WT FL protein construct and *TP53* mRNA (GelRed staining)

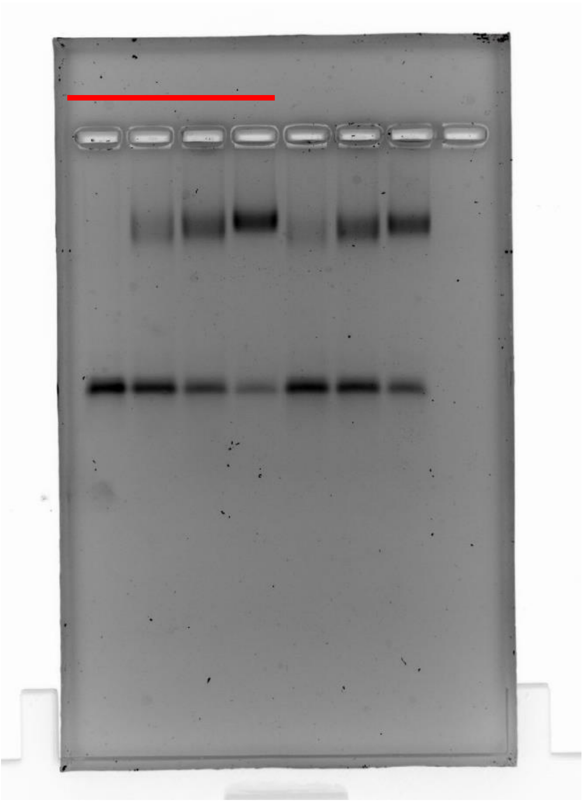

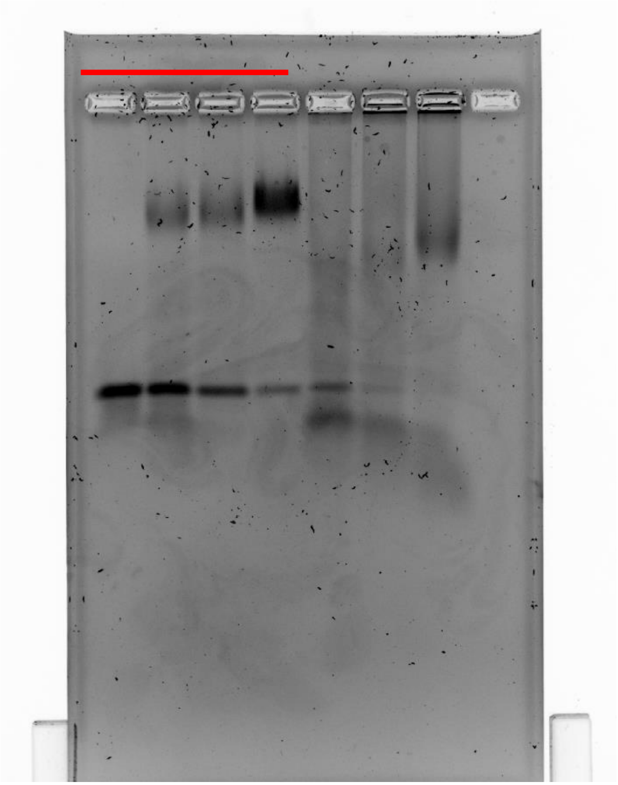

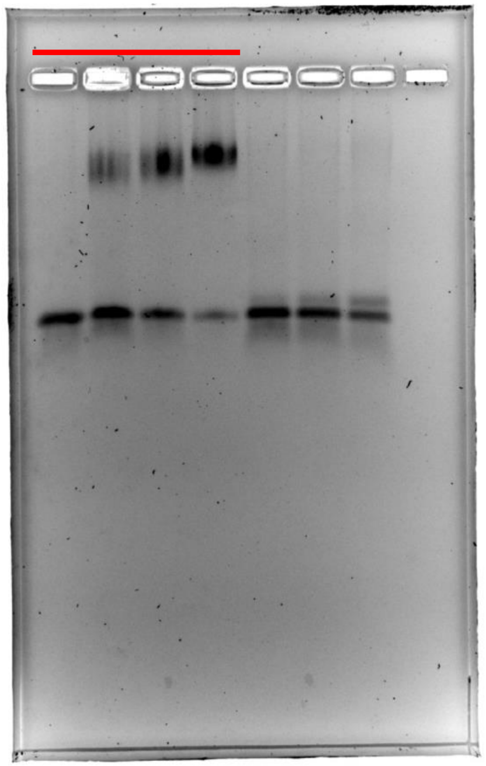

Lane assignment (gel lanes 1-4):
1 – RNA (ratio 1:0)
2 – RNA + MDMX WT FL (ratio 1:25)
3 – RNA + MDMX WT FL (ratio 1:50)
4 – RNA + MDMX WT FL (ratio 1:100)

Raw data for Figure 1G

EMSA = Interaction between MDMX WT (322-490) protein construct and *TP53* mRNA (GelRed staining)

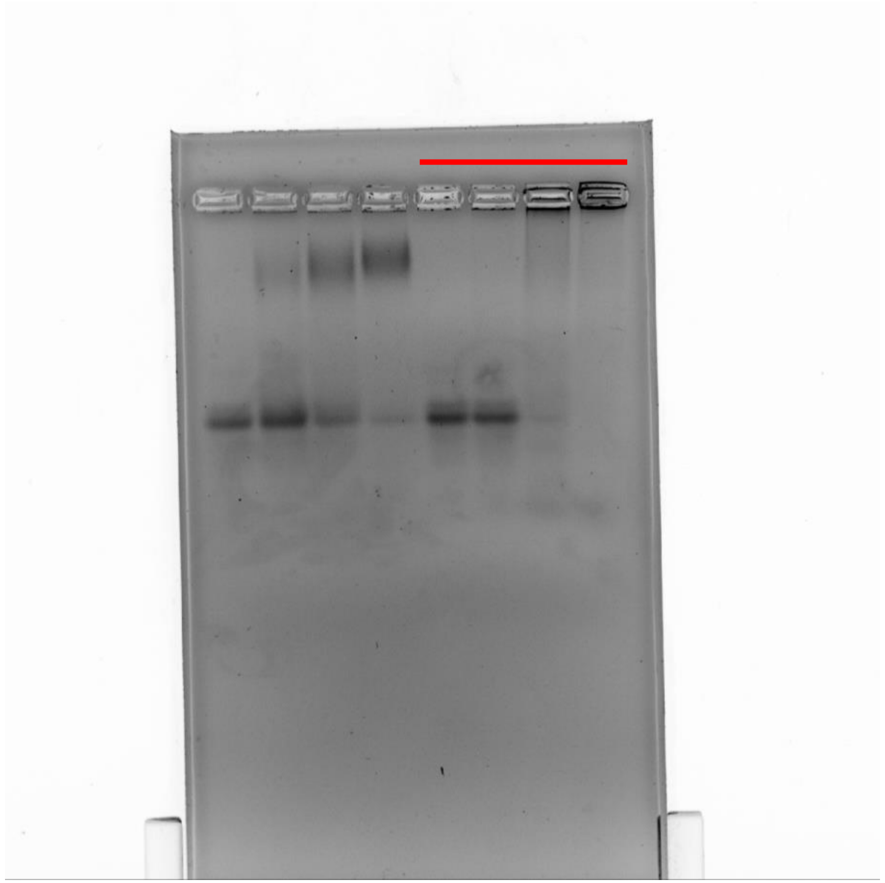

Lane assignment (gel lanes 5-8):
1 – RNA (ratio 1:0)
2 – RNA + MDMX WT (322-490) (ratio 1:25)
3 – RNA + MDMX WT (322-490) (ratio 1:50)
4 – RNA + MDMX WT (322-490) (ratio 1:100)

Replicates for Figure 1G

EMSA = Interaction between MDMX WT (322-490) protein construct and *TP53* mRNA (GelRed staining)

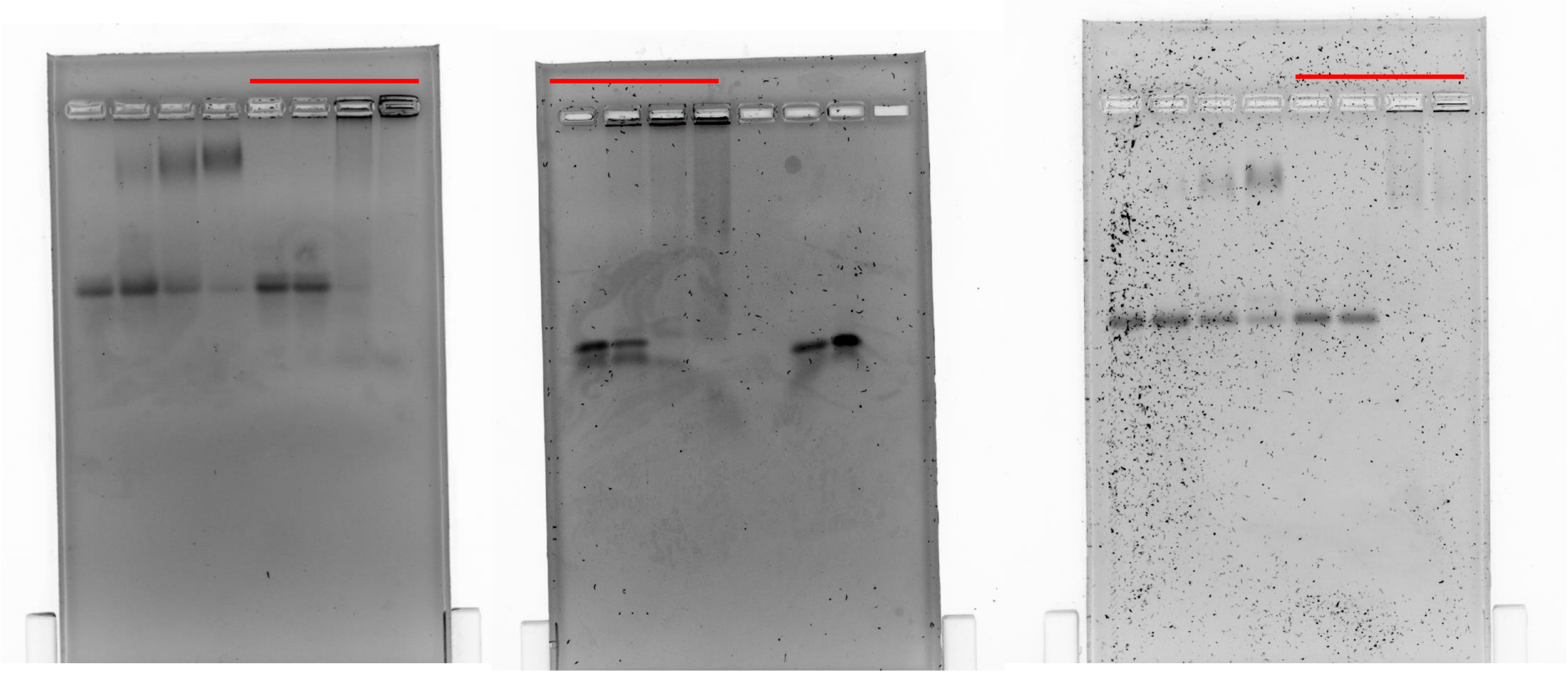

Lane assignment (gel lanes 5-8):

1 – RNA (ratio 1:0)

2 – RNA + MDMX WT (322-490) (ratio 1:25)

3 – RNA + MDMX WT (322-490) (ratio 1:50)

4 – RNA + MDMX WT (322-490) (ratio 1:100)

Raw data for Figure 2B

EMSA = Effect of NaCl on the interaction between MDMX-S403D (322-490) and *TP53* mRNA (GelRed staining)

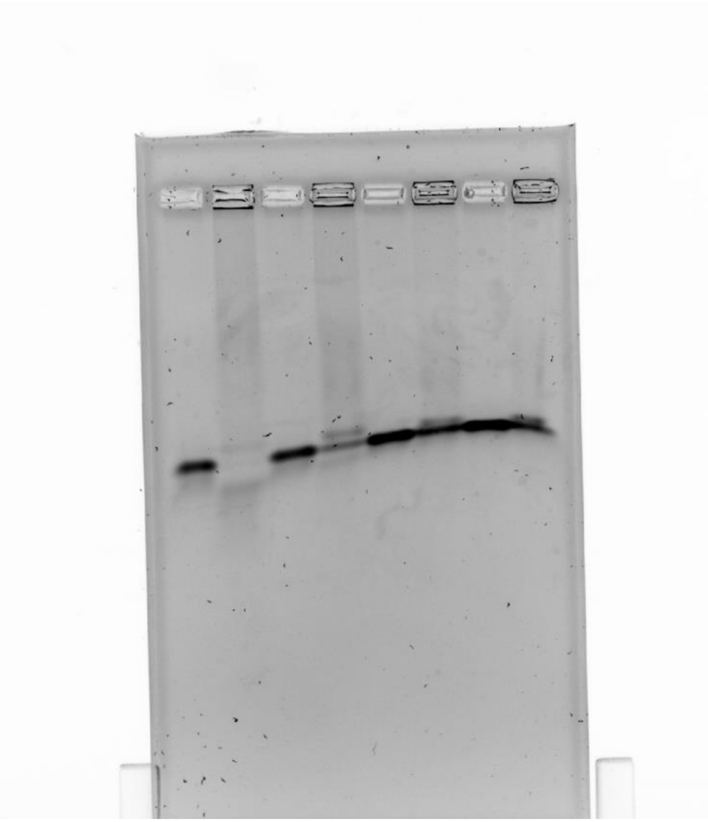

Lane assignment:
1 – RNA + 150 mM NaCl
2 – RNA + MDMX-S403D (322-490) + 150 mM NaCl
3 – RNA + 250 mM NaCl
4 – RNA + MDMX-S403D (322-490) + 250 mM NaCl
5 - RNA + 400 mM NaCl
6 – RNA + MDMX-S403D (322-490) + 400 mM NaCl
7 - RNA + 600 mM NaCl
8 - RNA + MDMX-S403D (322-490) + 600 mM NaCl

Replicates for Figure 2B

EMSA = Effect of NaCl on the interaction between MDMX-S403D (322-490) and *TP53* mRNA (GelRed staining)

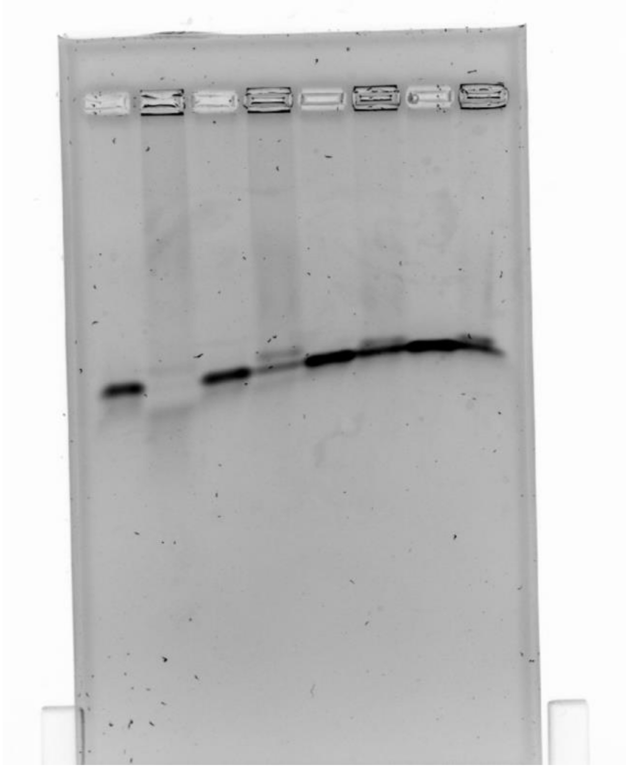

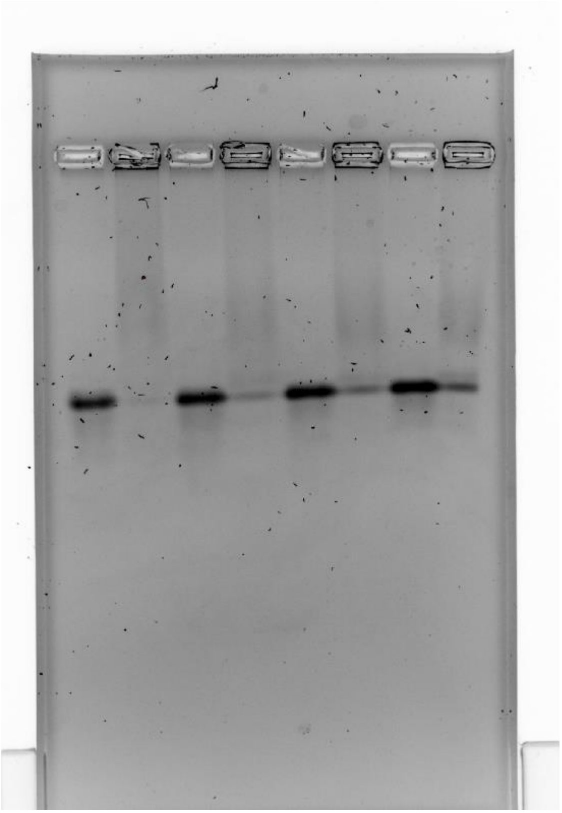

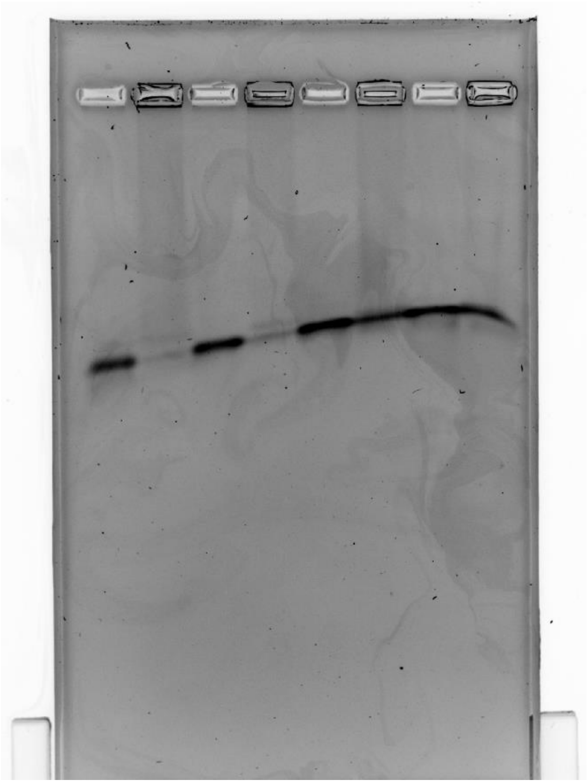

Lane assignment:

1 – RNA + 150 mM NaCl
2 – RNA + MDMX-S403D (322-490) + 150 mM NaCl
3 – RNA + 250 mM NaCl
4 – RNA + MDMX-S403D (322-490) + 250 mM NaCl
5 - RNA + 400 mM NaCl
6 – RNA + MDMX-S403D (322-490) + 400 mM NaCl
7 - RNA + 600 mM NaCl
8 - RNA + MDMX-S403D (322-490) + 600 mM NaCl

Raw data for Figure 2C

EMSA = Effect of NaCl on the interaction between MDMX-S403D FL and mRNA *TP53* (GelRed staining)

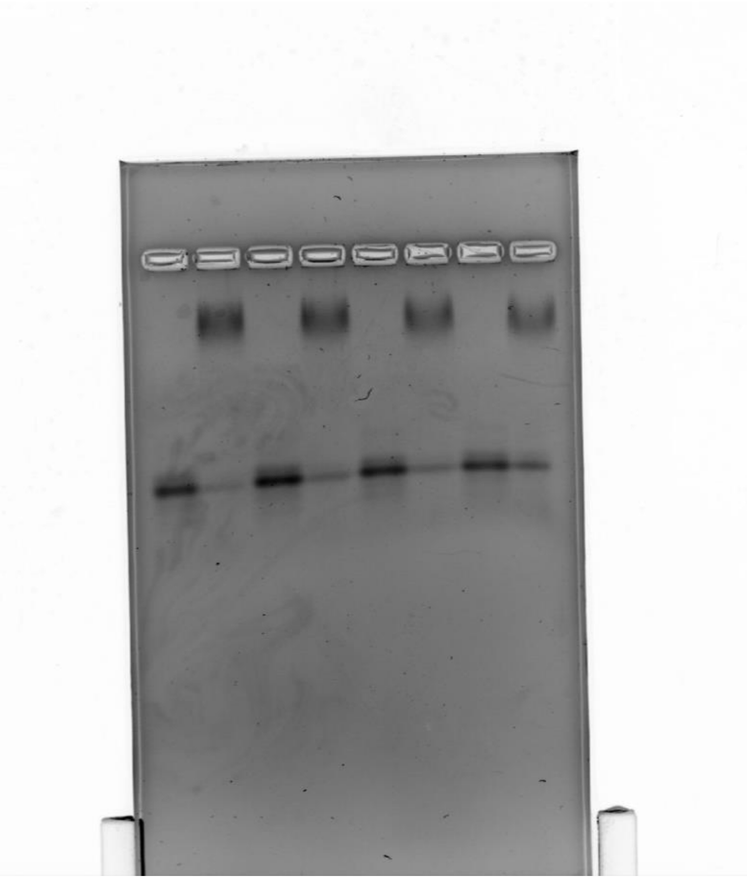

Lane assignment:
1 – RNA + 150 mM NaCl
2 – RNA + MDMX-S403D FL + 150 mM NaCl
3 – RNA + 250 mM NaCl
4 – RNA + MDMX-S403D FL + 250 mM NaCl
5 - RNA + 400 mM NaCl
6 – RNA + MDMX-S403D FL + 400 mM NaCl
7 - RNA + 600 mM NaCl
8 - RNA + MDMX-S403D FL + 600 mM NaCl

Replicates for Figure 2C

EMSA = Effect of NaCl on the interaction between MDMX-S403D FL and mRNA *TP53* (GelRed staining)

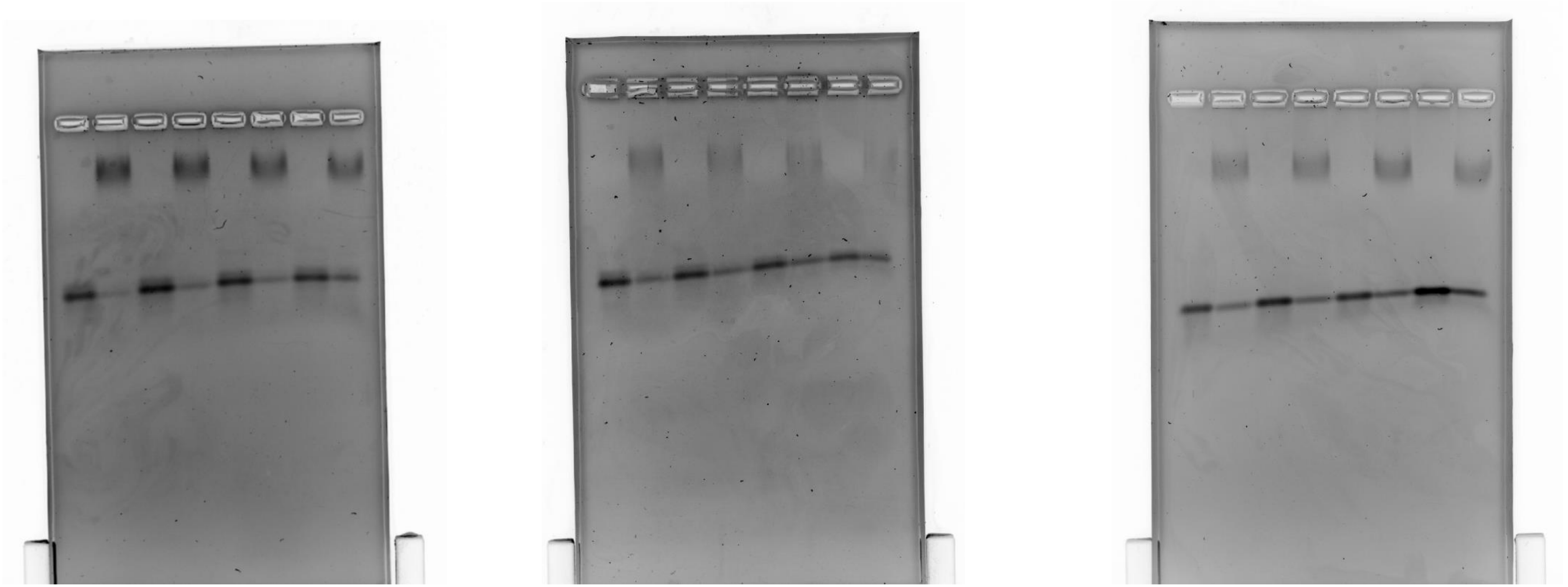

Lane assignment:

1 – RNA + 150 mM NaCl
2 – RNA + MDMX-S403D FL + 150 mM NaCl
3 – RNA + 250 mM NaCl
4 – RNA + MDMX-S403D FL + 250 mM NaCl
5 - RNA + 400 mM NaCl
6 – RNA + MDMX-S403D FL + 400 mM NaCl
7 - RNA + 600 mM NaCl
8 - RNA + MDMX-S403D FL + 600 mM NaCl

Raw data for Figure 3A

EMSA = Effect of pH on the interaction between MDMX-S403D FL and mRNA *TP53* (GelRed staining)

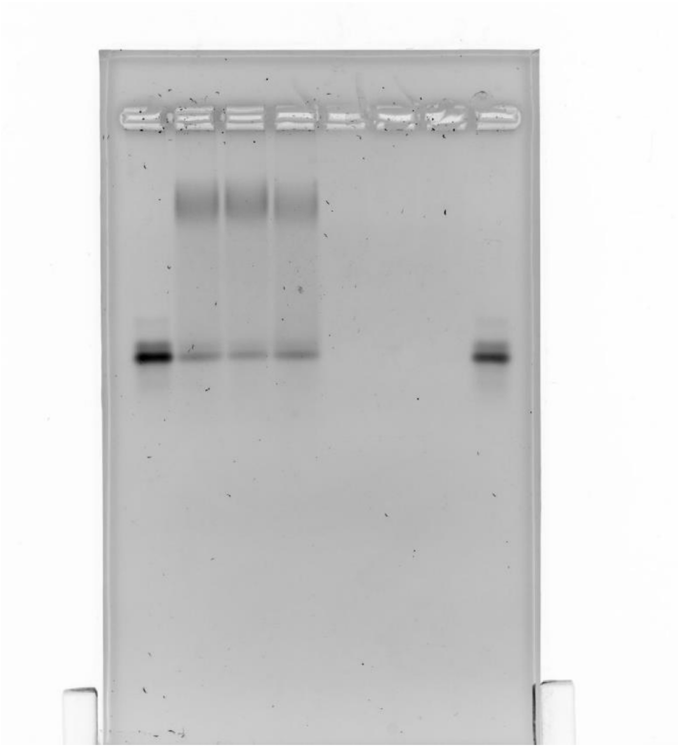

Lane assignment:
1 – RNA (pH 7.5)
2 – RNA + MDMX-S403D FL (pH 7.0)
3 – RNA + MDMX-S403D FL (pH 7.5)
4 – RNA + MDMX-S403D FL (pH 8.0)
5 - MDMX-S403D FL (pH 7.0)
6 – MDMX-S403D FL (pH 7.5)
7 - MDMX-S403D FL (pH 8.0)
8 - RNA (pH 8.0)

Raw data for Figure 3A

EMSA = Effect of pH on the interaction between MDMX-S403D FL and mRNA *TP53* (SYPRO Ruby staining)

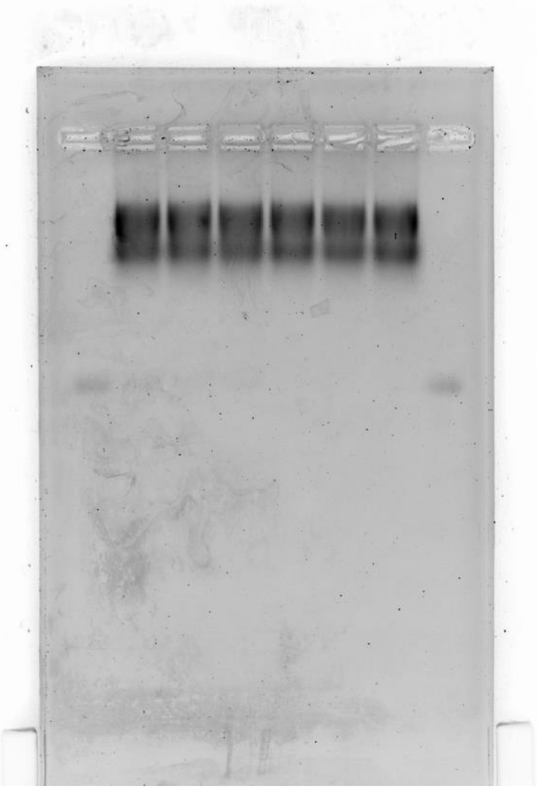

Lane assignment:
1 – RNA (pH 7.5)
2 – RNA + MDMX-S403D FL (pH 7.0)
3 – RNA + MDMX-S403D FL (pH 7.5)
4 – RNA + MDMX-S403D FL (pH 8.0)
5 - MDMX-S403D FL (pH 7.0)
6 – MDMX-S403D FL (pH 7.5)
7 - MDMX-S403D FL (pH 8.0)
8 - RNA (pH 8.0)

Replicates for Figure 3A

EMSA = Effect of pH on the interaction between MDMX-S403D FL and mRNA *TP53* (GelRed staining)

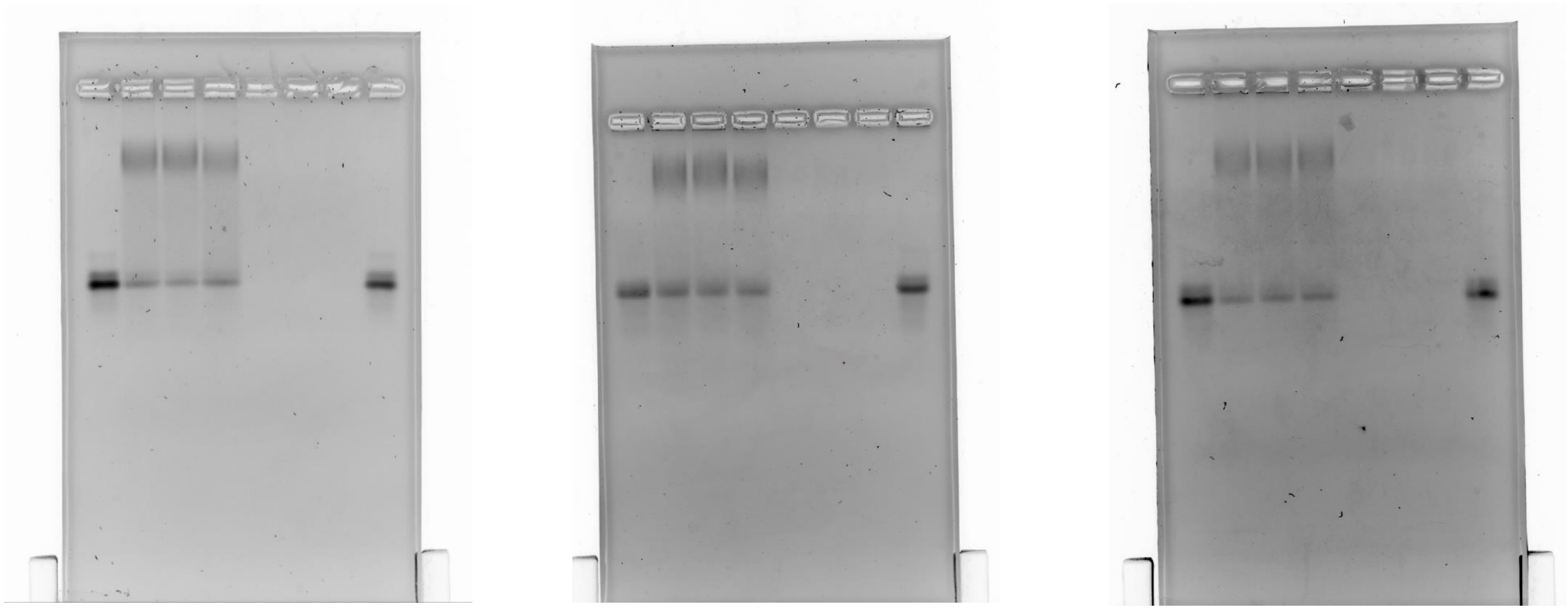

Lane assignment:

1 – RNA (pH 7.5)
2 – RNA + MDMX-S403D FL (pH 7.0)
3 – RNA + MDMX-S403D FL (pH 7.5)
4 – RNA + MDMX-S403D FL (pH 8.0)

5 - MDMX-S403D FL (pH 7.0)
6 – MDMX-S403D FL (pH 7.5)
7 - MDMX-S403D FL (pH 8.0)
8 - RNA (pH 8.0)

Raw data for Figure 3B

EMSA = Effect of pH on the interaction between MDMX-S403D (322-490) and mRNA *TP53* (GelRed staining)

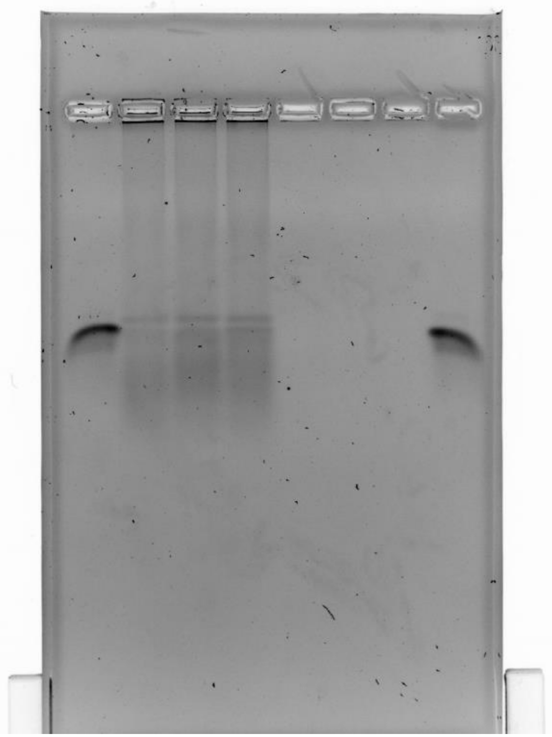

Lane assignment:
1 – RNA (pH 7.5)
2 – RNA + MDMX-S403D (322-490) (pH 7.0)
3 – RNA + MDMX-S403D (322-490) (pH 7.5)
4 – RNA + MDMX-S403D (322-490) (pH 8.0)
5 - MDMX-S403D (322-490) (pH 7.0)
6 – MDMX-S403D (322-490) (pH 7.5)
7 - MDMX-S403D (322-490) (pH 8.0)
8 - RNA (pH 8.0)

Raw data for Figure 3B

EMSA = Effect of pH on the interaction between MDMX-S403D (322-490) and mRNA *TP53* (SYPRO Ruby staining)

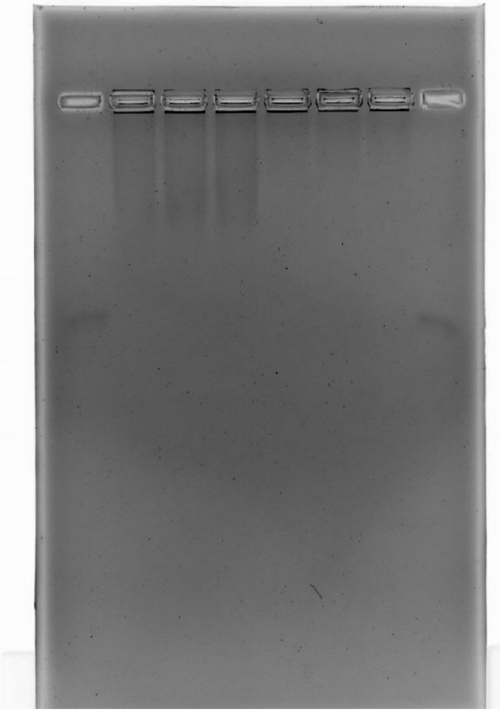

Lane assignment:
1 – RNA (pH 7.5)
2 – RNA + MDMX-S403D (322-490) (pH 7.0)
3 – RNA + MDMX-S403D (322-490) (pH 7.5)
4 – RNA + MDMX-S403D (322-490) (pH 8.0)
5 - MDMX-S403D (322-490) (pH 7.0)
6 – MDMX-S403D (322-490) (pH 7.5)
7 - MDMX-S403D (322-490) (pH 8.0)
8 - RNA (pH 8.0)

Raw data for Figure 3B

EMSA = Effect of pH on the interaction between MDMX-S403D (322-490) and mRNA *TP53* (GelRed staining)

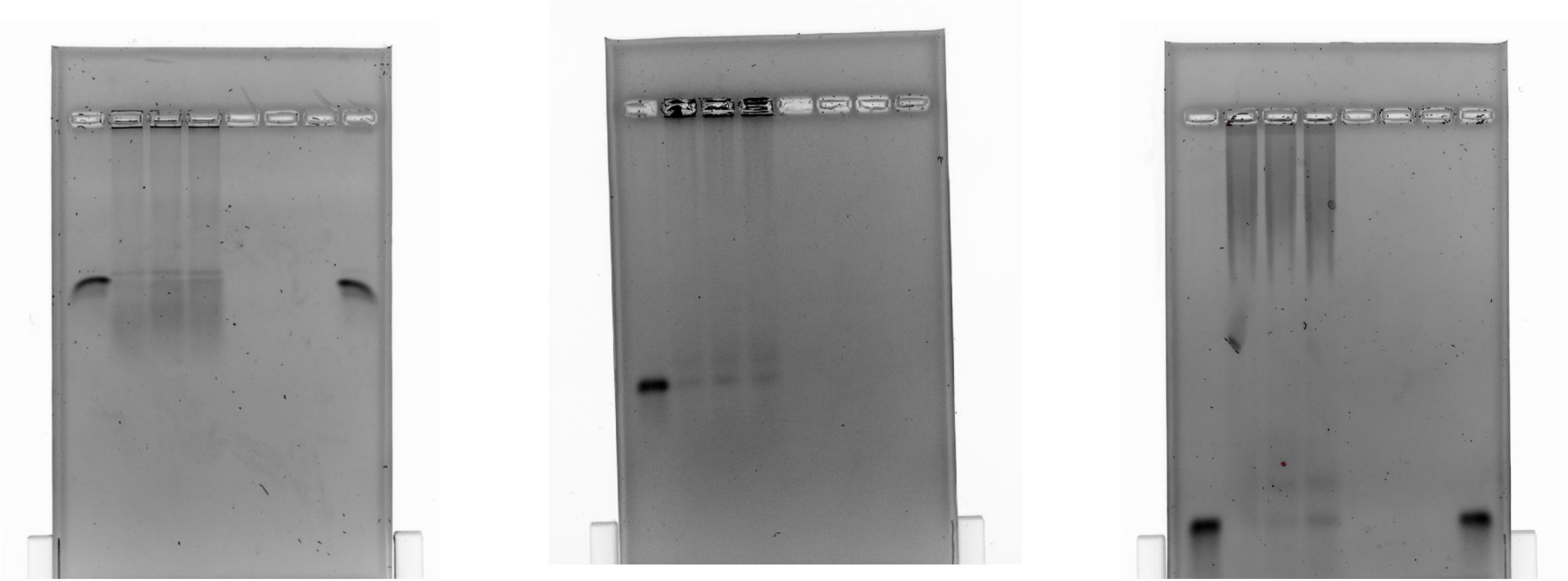

Lane assignment:

1 – RNA (pH 7.5)
2 – RNA + MDMX-S403D (322-490) (pH 7.0)
3 – RNA + MDMX-S403D (322-490) (pH 7.5)
4 – RNA + MDMX-S403D (322-490) (pH 8.0)
5 - MDMX-S403D (322-490) (pH 7.0)
6 – MDMX-S403D (322-490) (pH 7.5)
7 - MDMX-S403D (322-490) (pH 8.0)
8 - RNA (pH 8.0)

Raw data for Figure 4A

EMSA = Effect of $ZnSO_4$ and EDTA on the interaction between MDMX-S403D FL and mRNA *TP53* (GelRed staining)

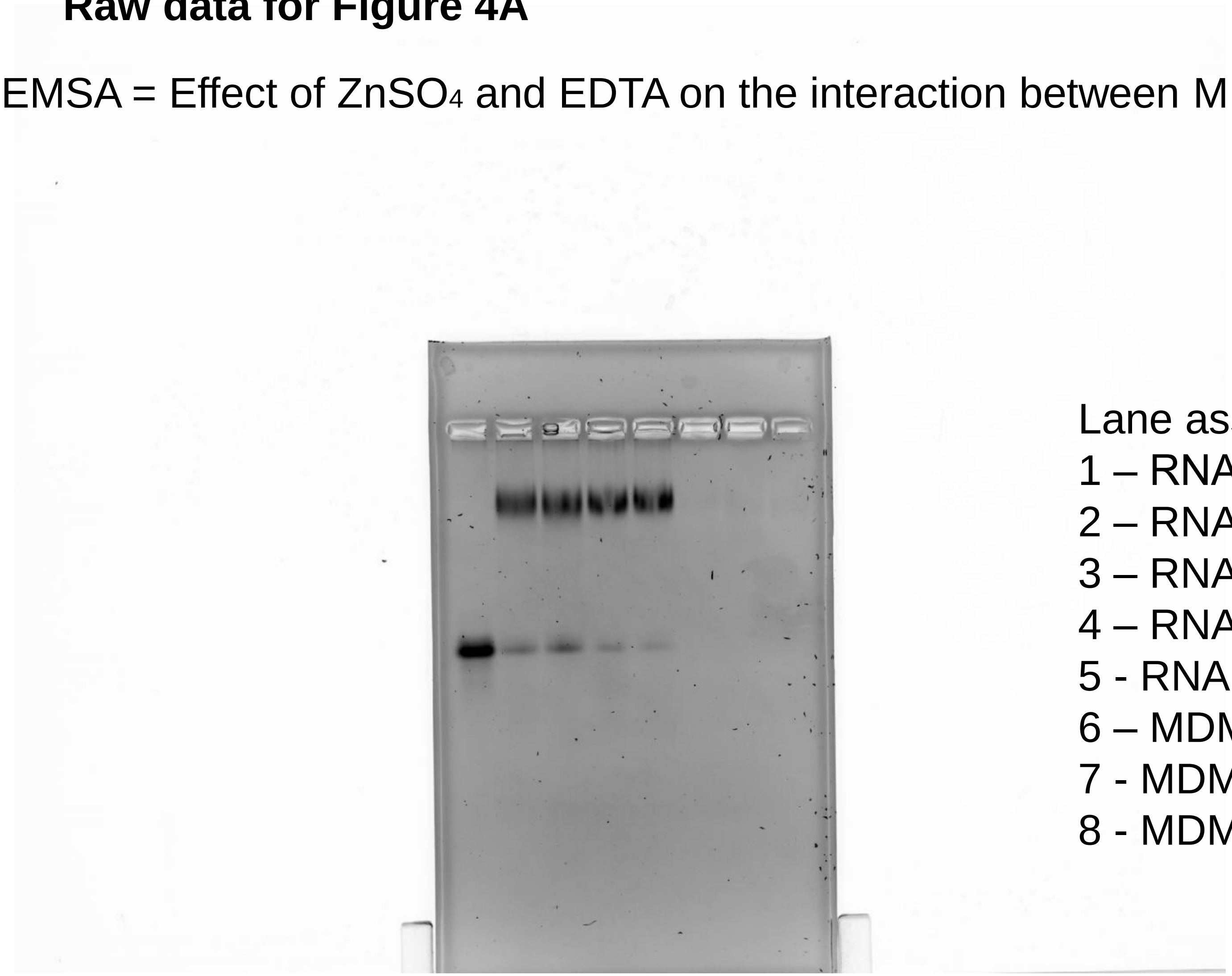

Lane assignment:
1 – RNA + 10 μM
2 – RNA + MDMX-S403D FL + 10 μM
3 – RNA + MDMX-S403D FL + 100 μM
4 – RNA + MDMX-S403D FL + 10 μM + EDTA
5 - RNA + MDMX-S403D FL + 100 μM + EDTA
6 – MDMX-S403D FL + 10 μM
7 - MDMX-S403D FL + 100 μM
8 - MDMX-S403D FL + 10 μM + EDTA

Raw data for Figure 4A

EMSA = Effect of ZnSO4 and EDTA on the interaction between MDMX-S403D FL and mRNA *TP53* (SYPRO Ruby staining)

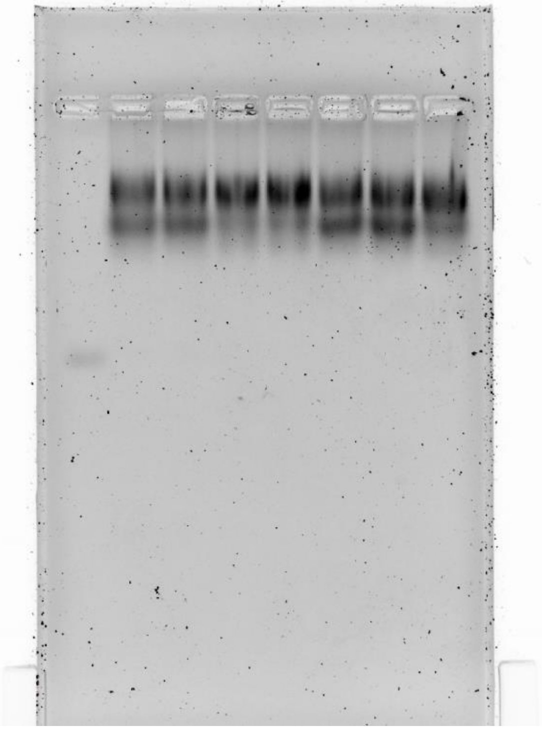

Lane assignment:
1 – RNA + 10 µM
2 – RNA + MDMX-S403D FL + 10 µM
3 – RNA + MDMX-S403D FL + 100 µM
4 – RNA + MDMX-S403D FL + 10 µM + EDTA
5 - RNA + MDMX-S403D FL + 100 µM + EDTA
6 – MDMX-S403D FL + 10 µM
7 - MDMX-S403D FL + 100 µM
8 - MDMX-S403D FL + 10 µM + EDTA

Replicates for Figure 4A

EMSA = Effect of ZnSO4 and EDTA on the interaction between MDMX-S403D FL and mRNA *TP53* (GelRed staining)

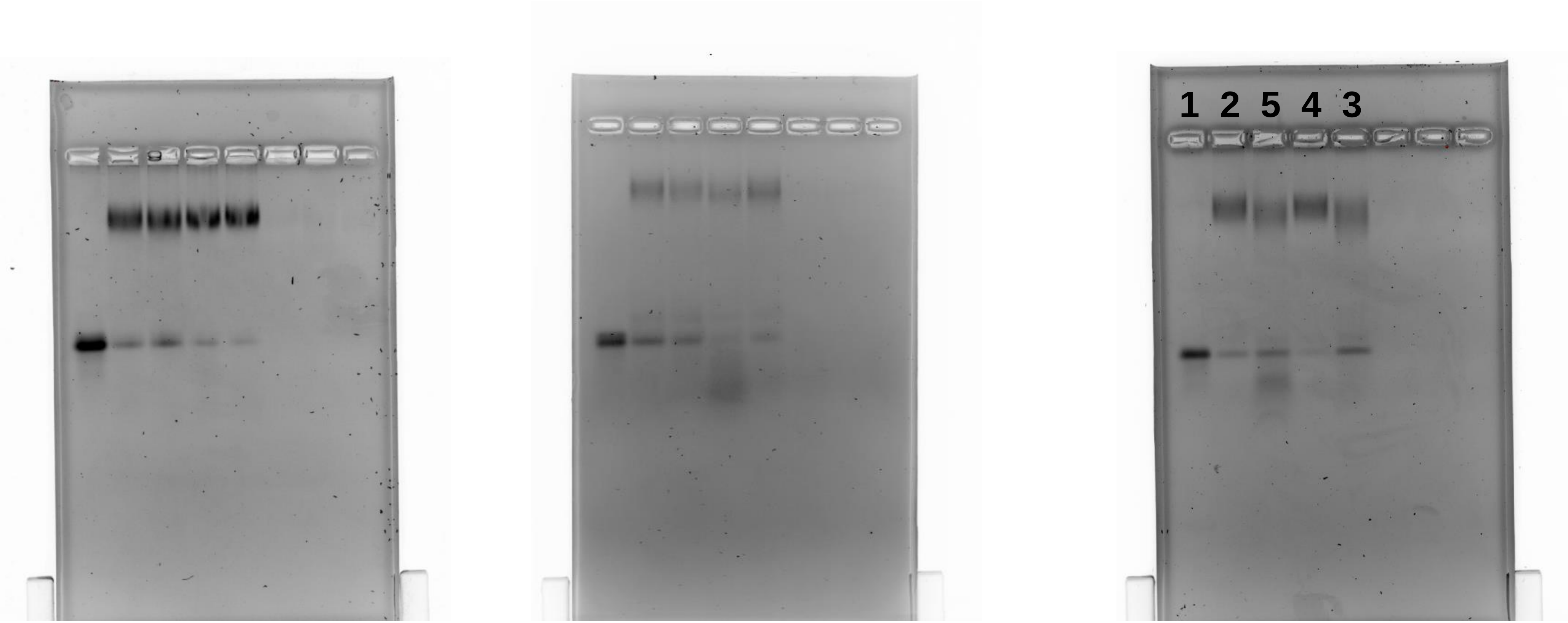

Lane assignment:

1 – RNA + 10 μM

2 – RNA + MDMX-S403D FL + 10 μM

3 – RNA + MDMX-S403D FL + 100 μM

4 – RNA + MDMX-S403D FL + 10 μM + EDTA

5 - RNA + MDMX-S403D FL + 100 μM + EDTA

6 – MDMX-S403D FL + 10 μM

7 - MDMX-S403D FL + 100 μM

8 - MDMX-S403D FL + 10 μM + EDTA

Raw data for Figure 4C

EMSA = Effect of TCEP on the interaction between MDMX-S403D FL and mRNA *TP53* (GelRed staining)

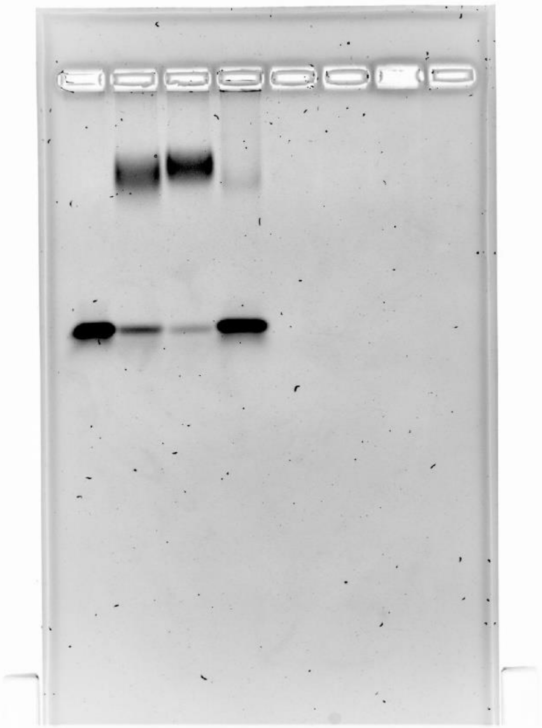

Lane assignment:
1 – RNA + 1 mM TCEP
2 – RNA + MDMX-S403D FL
3 – RNA + MDMX-S403D FL + 1 mM TCEP
4 – RNA + MDMX-S403D FL + 5 mM TCEP
5 - MDMX-S403D FL
6 – MDMX-S403D FL + 1 mM TCEP
7 - MDMX-S403D FL + 5 mM TCEP

Raw data for Figure 4C

EMSA = Effect of TCEP on the interaction between MDMX-S403D FL and mRNA *TP53* (SYPRO Ruby staining)

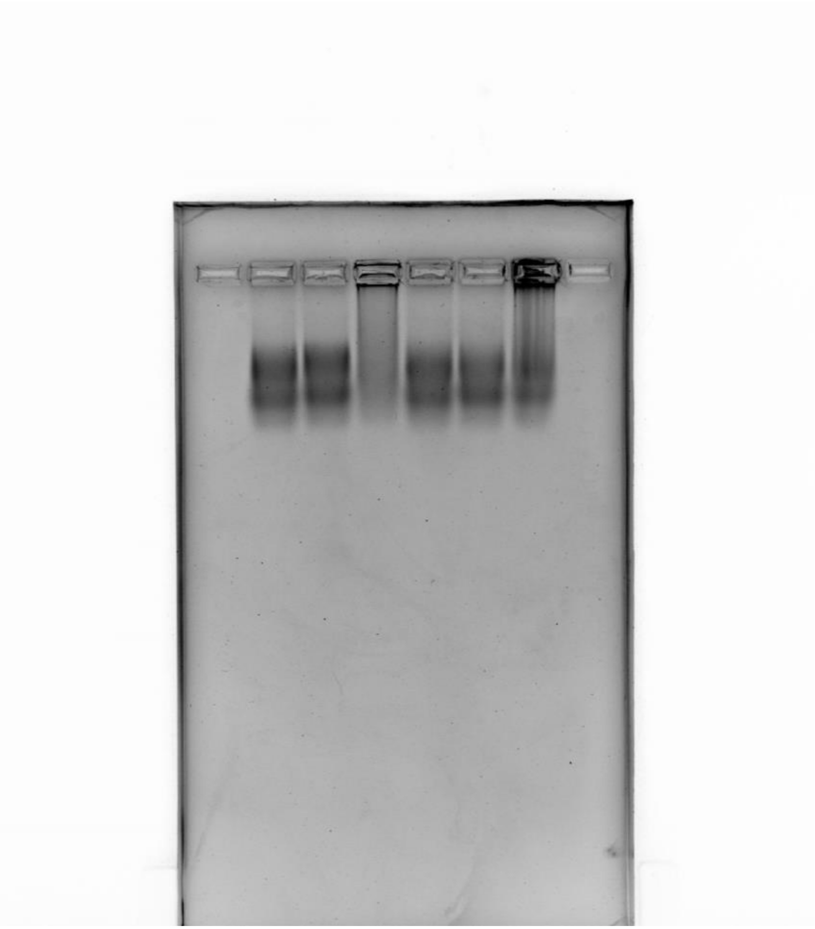

Lane assignment:
1 – RNA + 1 mM TCEP
2 – RNA + MDMX-S403D FL
3 – RNA + MDMX-S403D FL + 1 mM TCEP
4 – RNA + MDMX-S403D FL + 5 mM TCEP
5 - MDMX-S403D FL
6 – MDMX-S403D FL + 1 mM TCEP
7 - MDMX-S403D FL + 5 mM TCEP

Replicates for Figure 4C

EMSA = Effect of TCEP on the interaction between MDMX-S403D FL and mRNA *TP53* (GelRed staining)

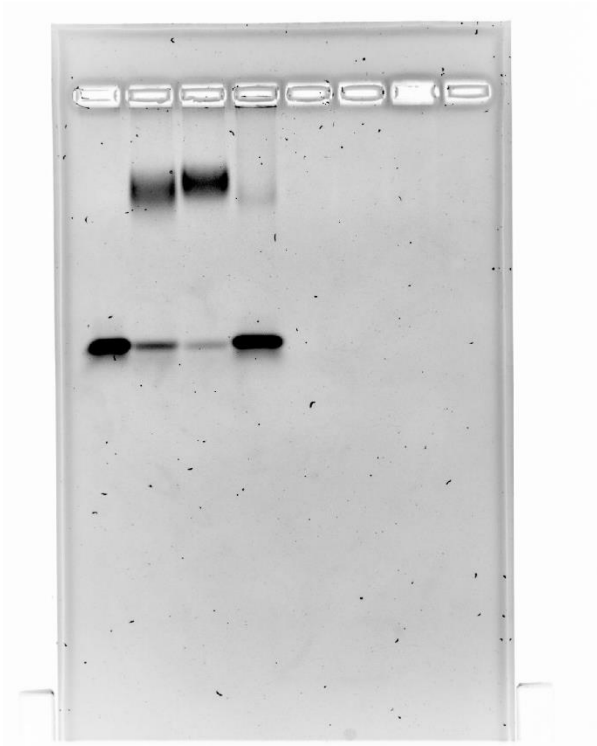

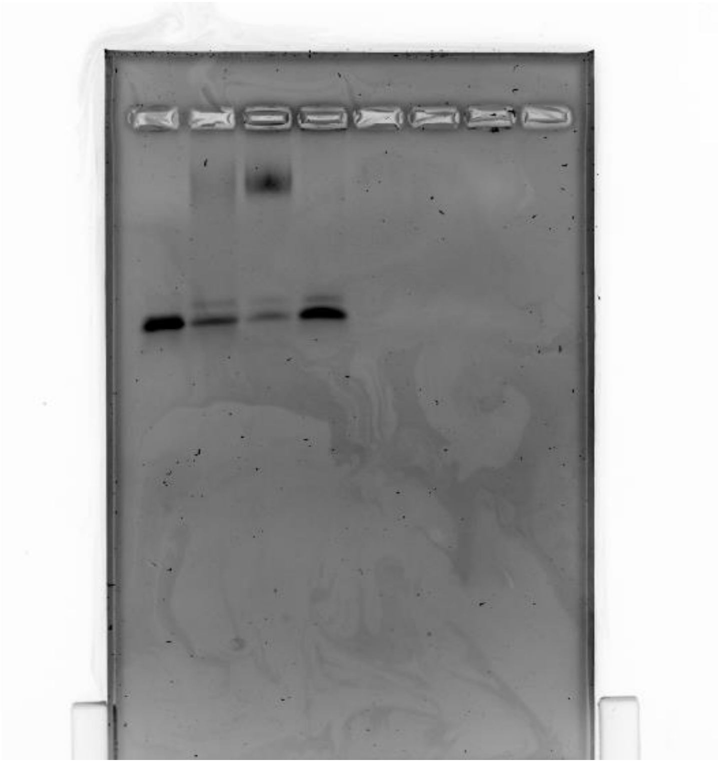

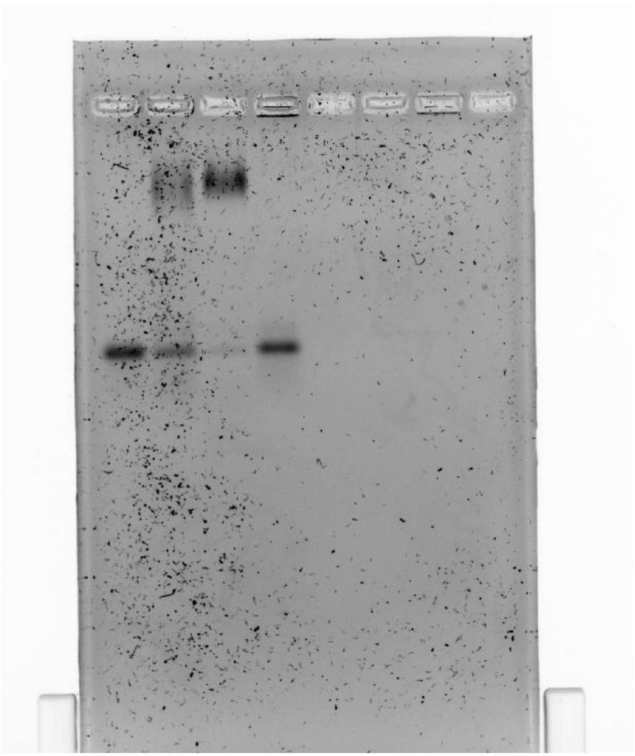

Lane assignment:
1 – RNA + 1 mM TCEP
2 – RNA + MDMX-S403D FL
3 – RNA + MDMX-S403D FL + 1 mM TCEP
4 – RNA + MDMX-S403D FL + 5 mM TCEP
5 - MDMX-S403D FL
6 – MDMX-S403D FL + 1 mM TCEP
7 - MDMX-S403D FL + 5 mM TCEP

Raw data for Figure S2

EMSA = Effect of NaCl on free mRNA *TP53* (GelRed staining)

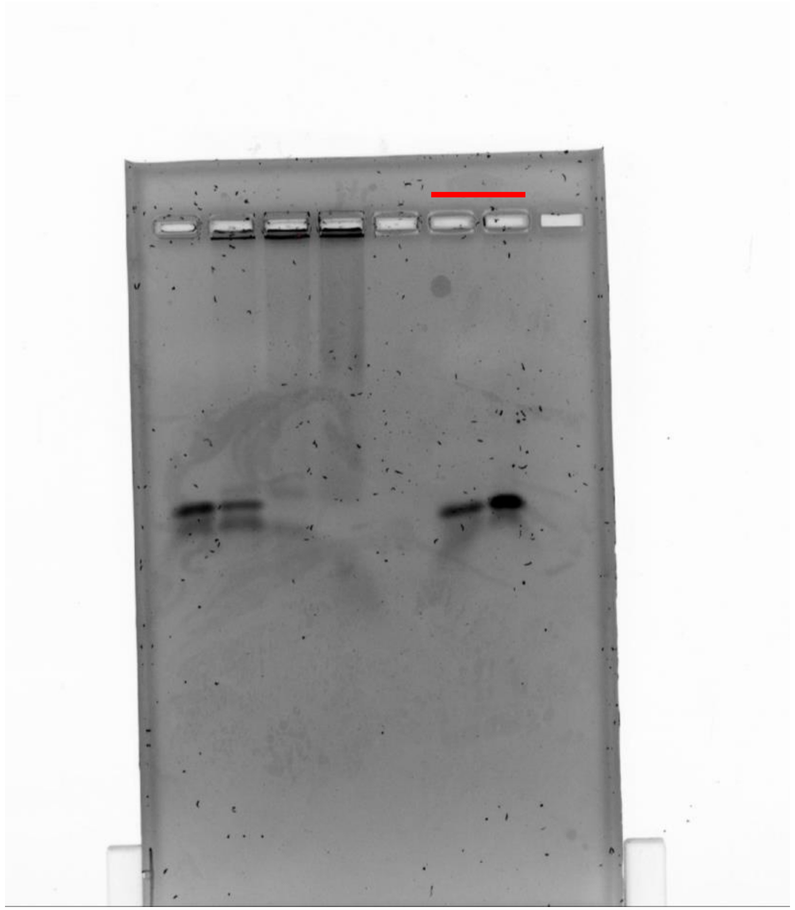

Lane assignment (gel lanes 6,7):
1 – RNA + 150 mM NaCl
2 – RNA + 600 mM NaCl

Raw data for Figure S4A

EMSA = Effect of ZnSO4 and EDTA on the interaction between MDMX-S403D (322-490) and mRNA *TP53* (GelRed staining)

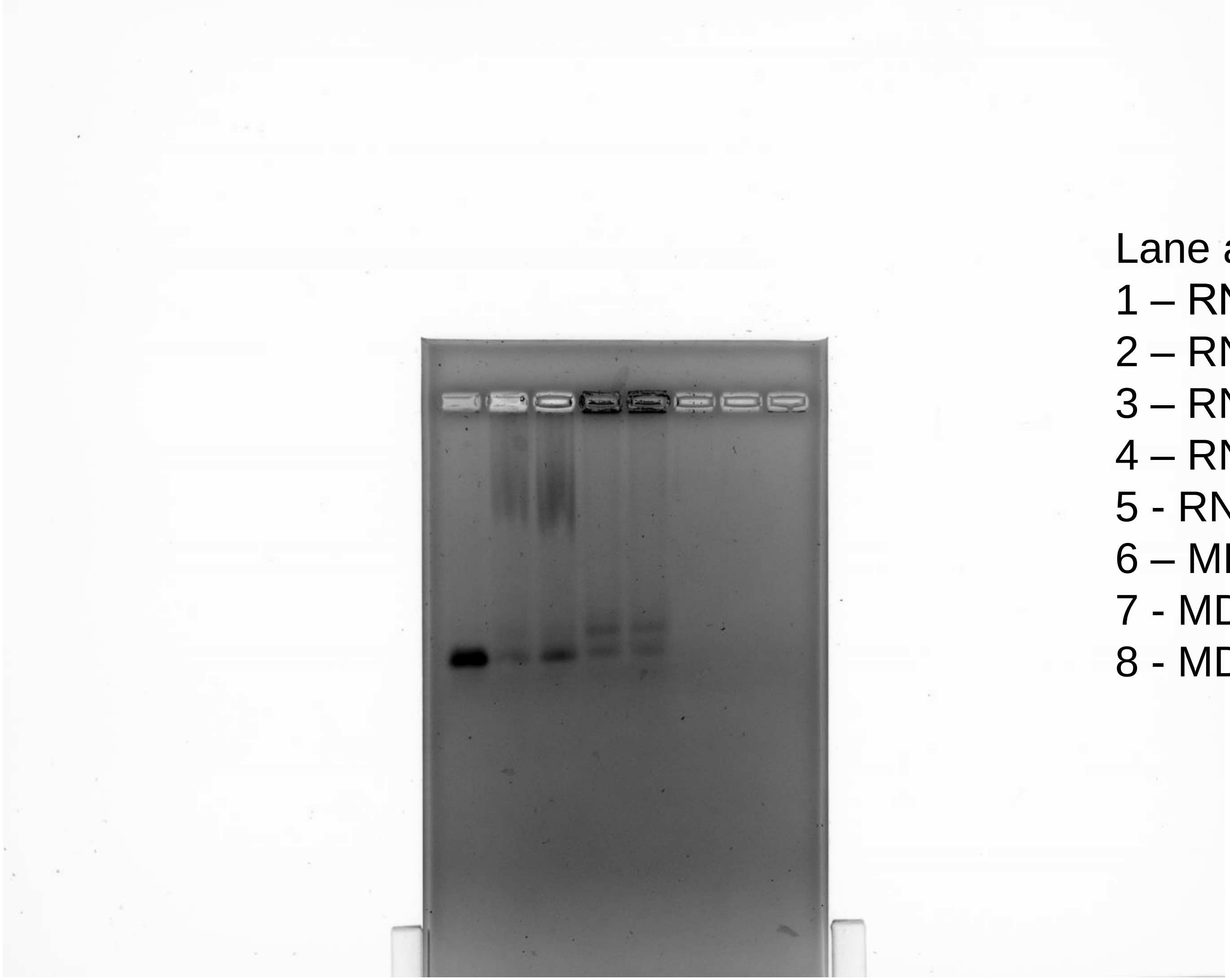

Lane assignment:
1 – RNA + 10 µM
2 – RNA + MDMX-S403D (322-490) + 10 µM
3 – RNA + MDMX-S403D (322-490) + 100 µM
4 – RNA + MDMX-S403D (322-490) + 10 µM + EDTA
5 - RNA + MDMX-S403D (322-490) + 100 µM + EDTA
6 – MDMX-S403D (322-490) + 10 µM
7 - MDMX-S403D (322-490) + 100 µM
8 - MDMX-S403D (322-490) + 10 µM + EDTA

Raw data for Figure S4A

EMSA = Effect of $ZnSO_4$ and EDTA on the interaction between MDMX-S403D (322-490) and mRNA *TP53* (SYPRO Ruby staining)

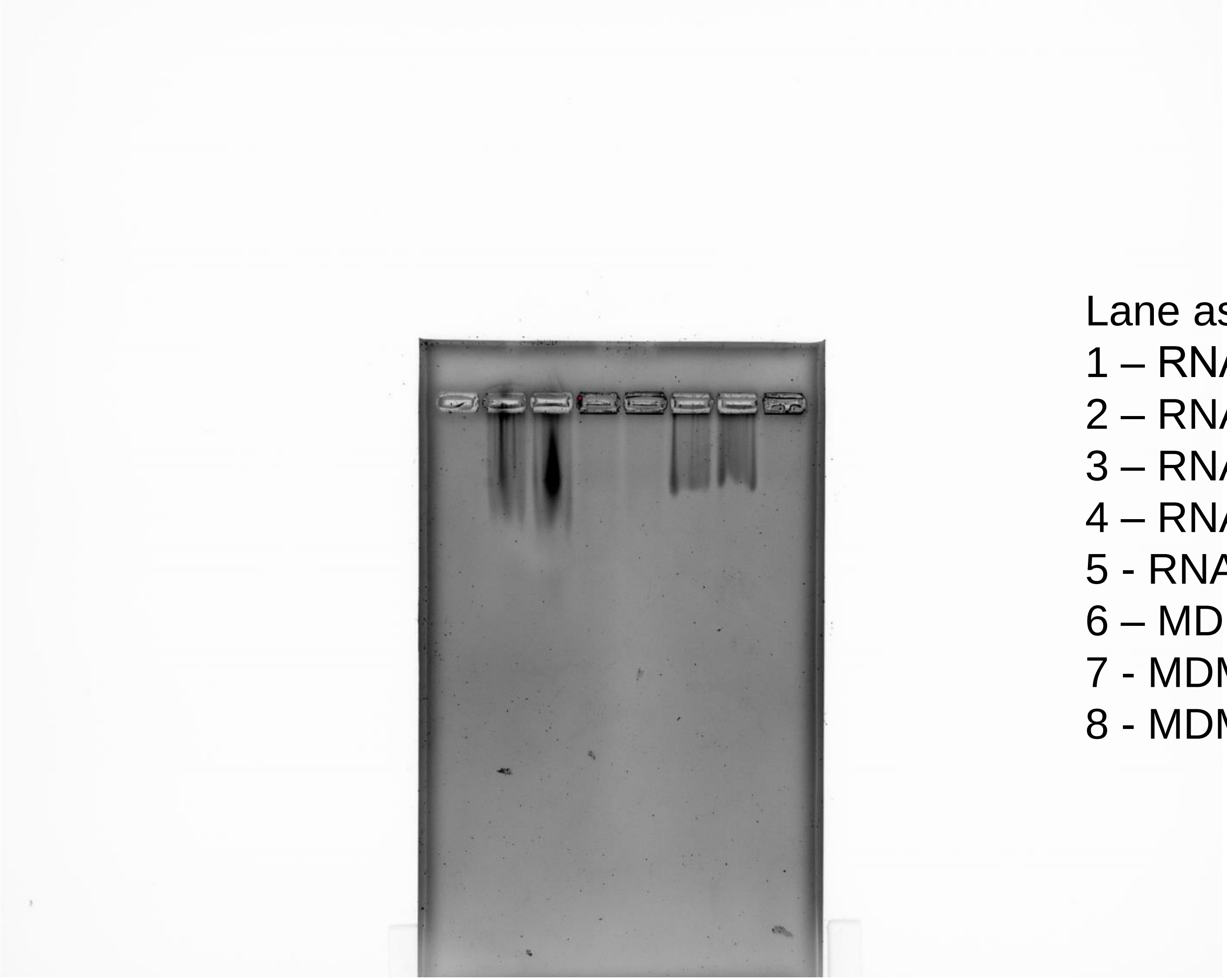

Lane assignment:

1 – RNA + 10 µM

2 – RNA + MDMX-S403D (322-490) + 10 µM

3 – RNA + MDMX-S403D (322-490) + 100 µM

4 – RNA + MDMX-S403D (322-490) + 10 µM + EDTA

5 - RNA + MDMX-S403D (322-490) + 100 µM + EDTA

6 – MDMX-S403D (322-490) + 10 µM

7 - MDMX-S403D (322-490) + 100 µM

8 - MDMX-S403D (322-490) + 10 µM + EDTA

Raw data for Figure S4A

EMSA = Effect of ZnSO4 and EDTA on the interaction between MDMX-S403D (322-490) and mRNA *TP53* (GelRed staining)

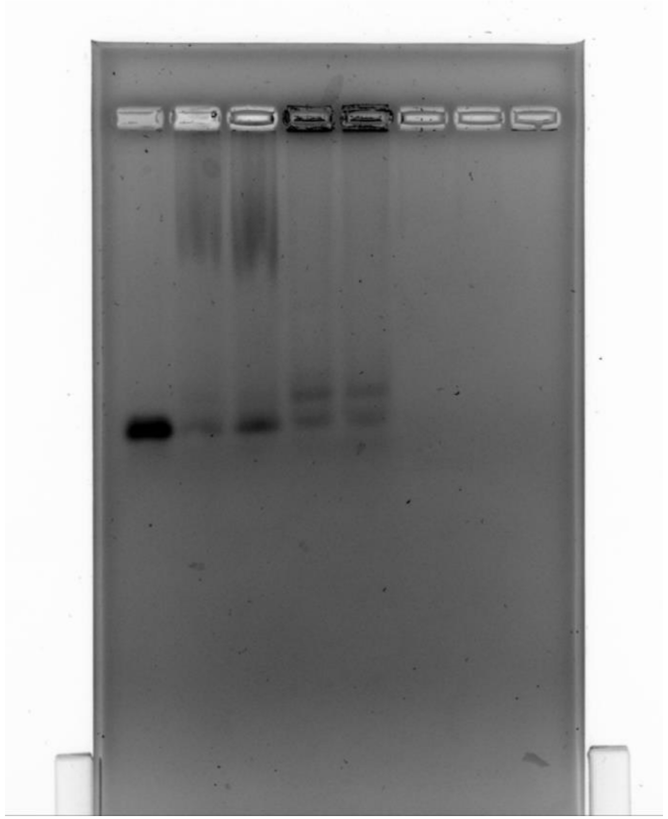

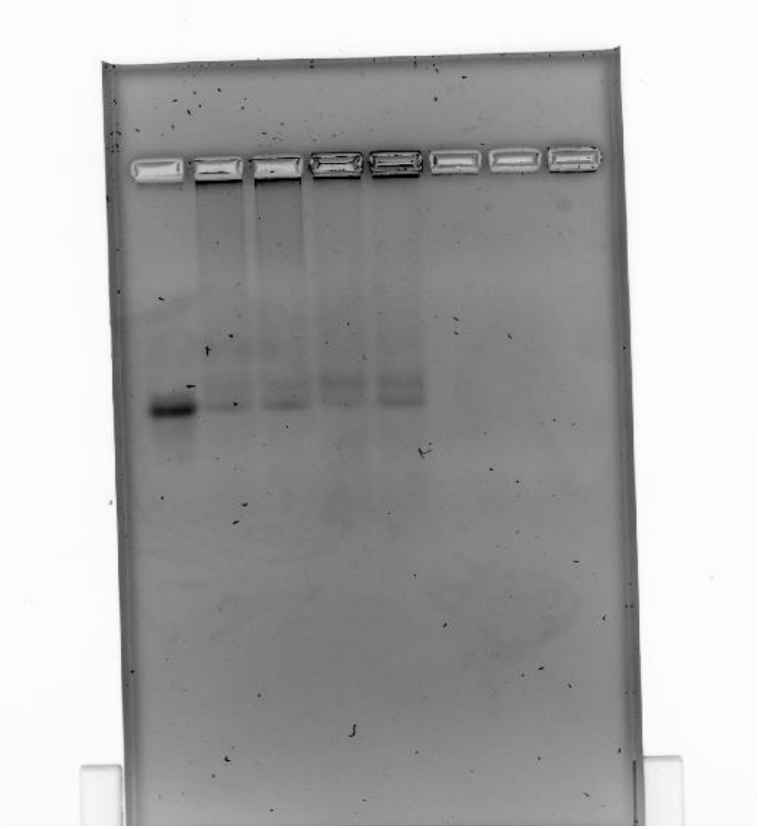

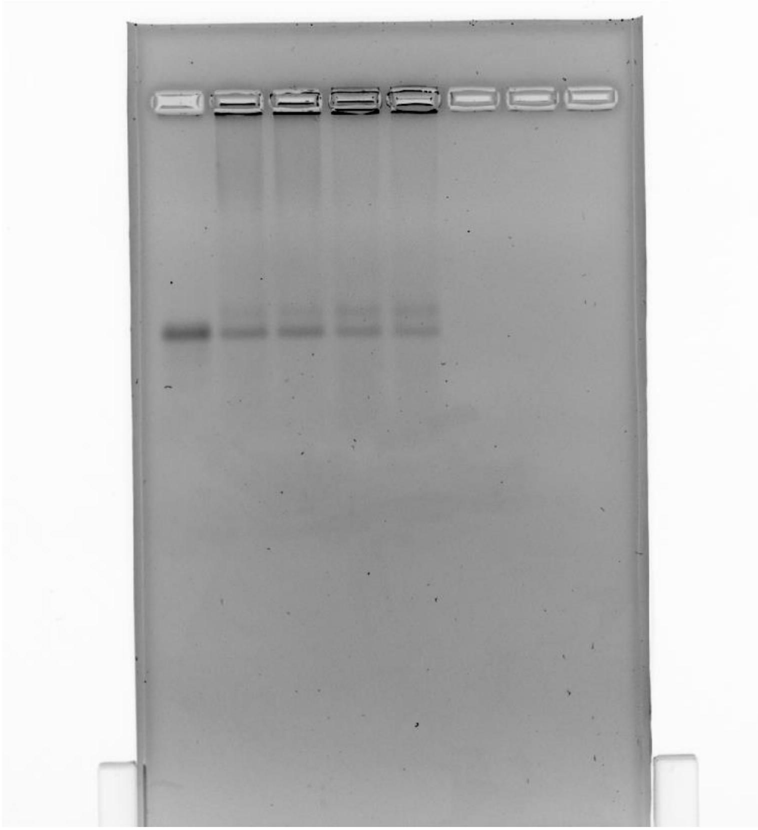

Lane assignment:

1 – RNA + 10 µM
2 – RNA + MDMX-S403D (322-490) + 10 µM
3 – RNA + MDMX-S403D (322-490) + 100 µM
4 – RNA + MDMX-S403D (322-490) + 10 µM + EDTA
5 - RNA + MDMX-S403D (322-490) + 100 µM + EDTA
6 – MDMX-S403D (322-490) + 10 µM
7 - MDMX-S403D (322-490) + 100 µM
8 - MDMX-S403D (322-490) + 10 µM + EDTA

Raw data for Figure S4C

EMSA = Effect of TCEP on the interaction between MDMX-S403D (322-490) and mRNA *TP53* (GelRed staining)

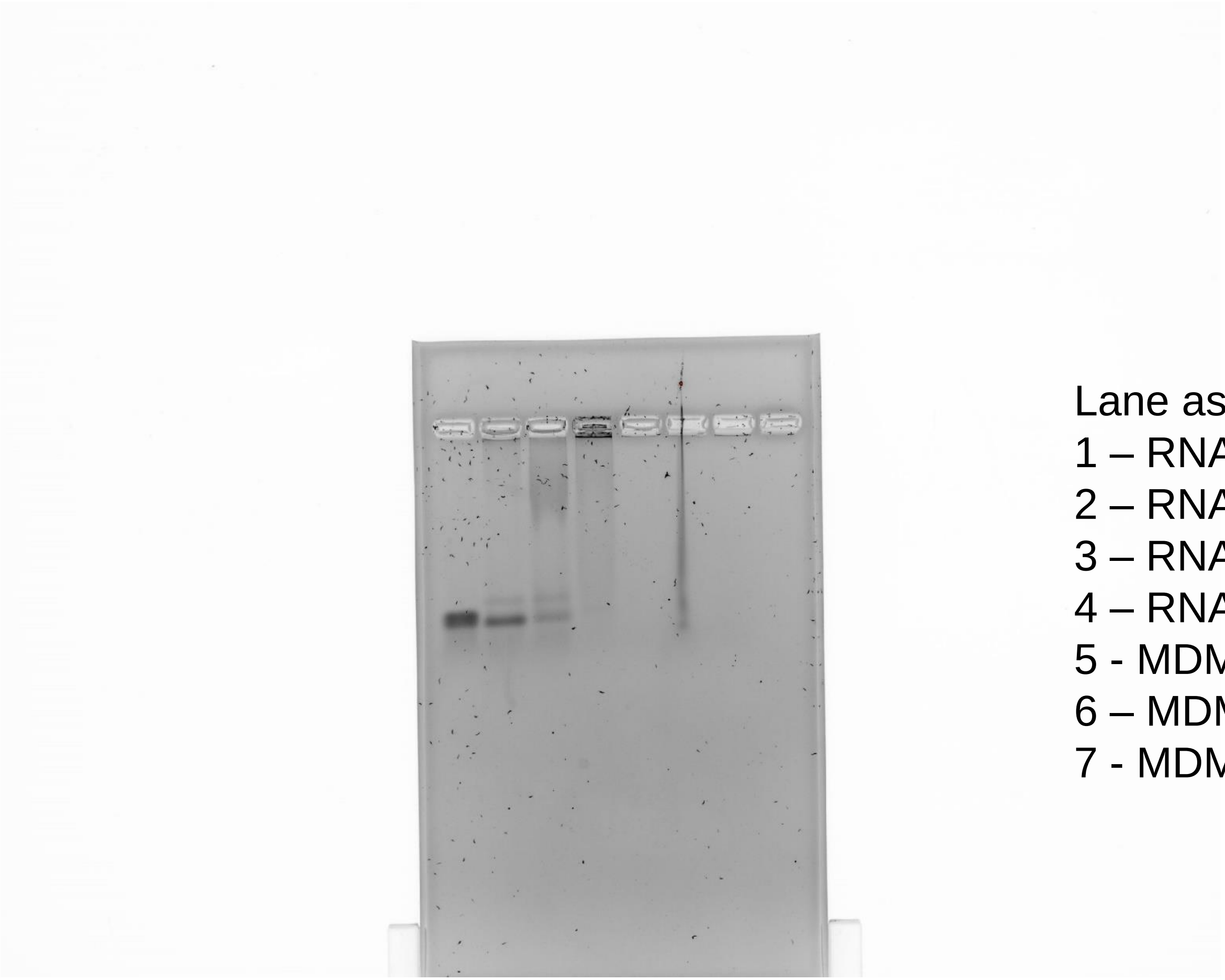

Lane assignment:
1 – RNA + 1 mM TCEP
2 – RNA + MDMX-S403D (322-490)
3 – RNA + MDMX-S403D (322-490) + 1 mM TCEP
4 – RNA + MDMX-S403D (322-490) + 5 mM TCEP
5 - MDMX-S403D (322-490)
6 – MDMX-S403D (322-490) + 1 mM TCEP
7 - MDMX-S403D (322-490) + 5 mM TCEP

Raw data for Figure S4C

EMSA = Effect of TCEP on the interaction between MDMX-S403D (322-490) and mRNA *TP53* (SYPRO Ruby staining)

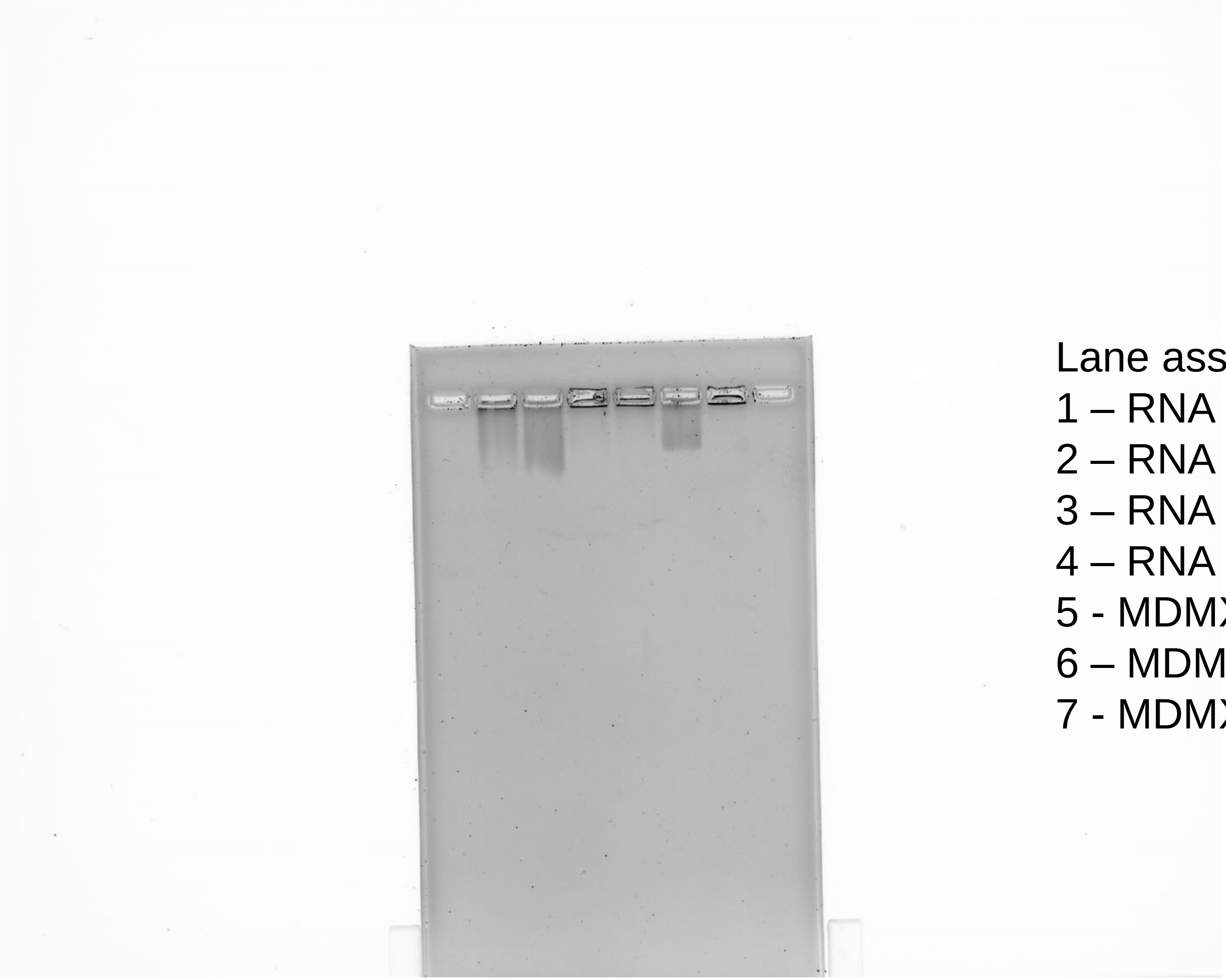

Lane assignment:
1 – RNA + 1 mM TCEP
2 – RNA + MDMX-S403D (322-490)
3 – RNA + MDMX-S403D (322-490) + 1 mM TCEP
4 – RNA + MDMX-S403D (322-490) + 5 mM TCEP
5 - MDMX-S403D (322-490)
6 – MDMX-S403D (322-490) + 1 mM TCEP
7 - MDMX-S403D (322-490) + 5 mM TCEP

Raw data for Figure S4C

EMSA = Effect of TCEP on the interaction between MDMX-S403D (322-490) and mRNA *TP53* (GelRed staining)

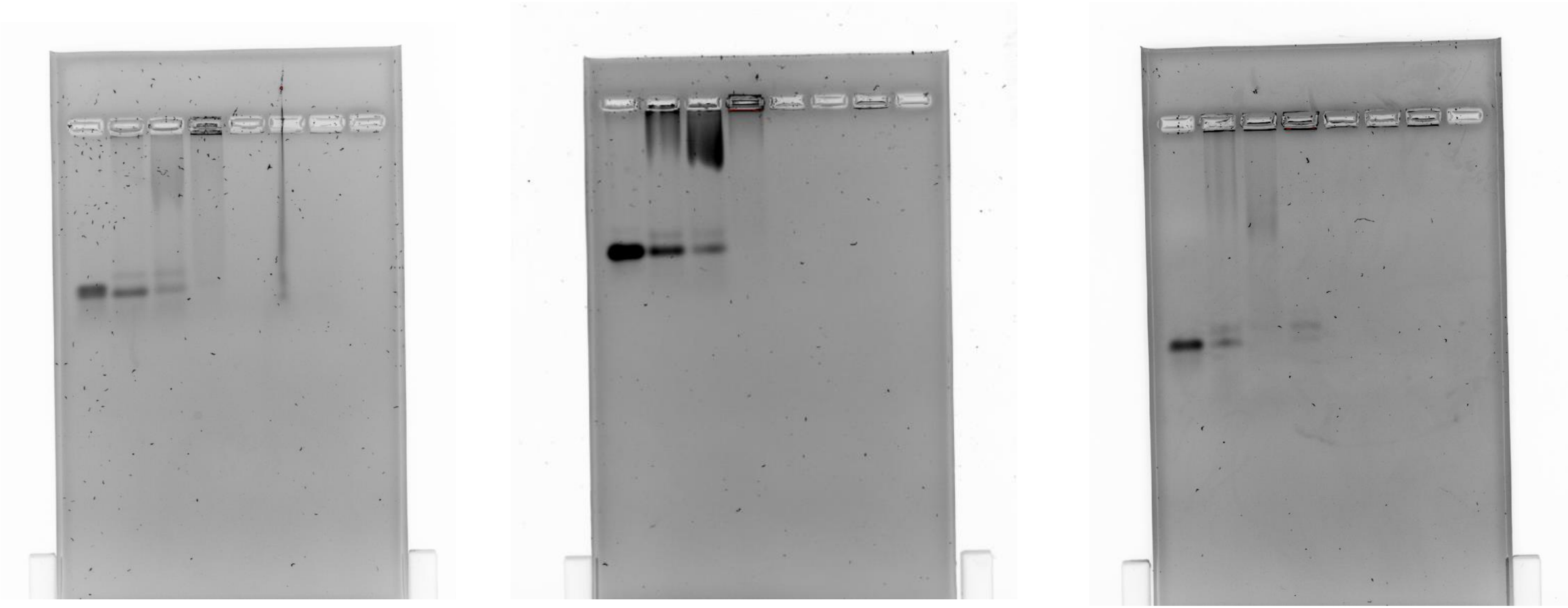

Lane assignment:
1 – RNA + 1 mM TCEP
2 – RNA + MDMX-S403D (322-490)
3 – RNA + MDMX-S403D (322-490) + 1 mM TCEP
4 – RNA + MDMX-S403D (322-490) + 5 mM TCEP
5 - MDMX-S403D (322-490)
6 – MDMX-S403D (322-490) + 1 mM TCEP
7 - MDMX-S403D (322-490) + 5 mM TCEP

Raw data for Figure S4E

EMSA = Effect of $ZnSO_4$ and EDTA on the interaction between MDMX-S403D FL and mRNA *TP53* (overlay of GelRed and SYPRO Ruby staining)

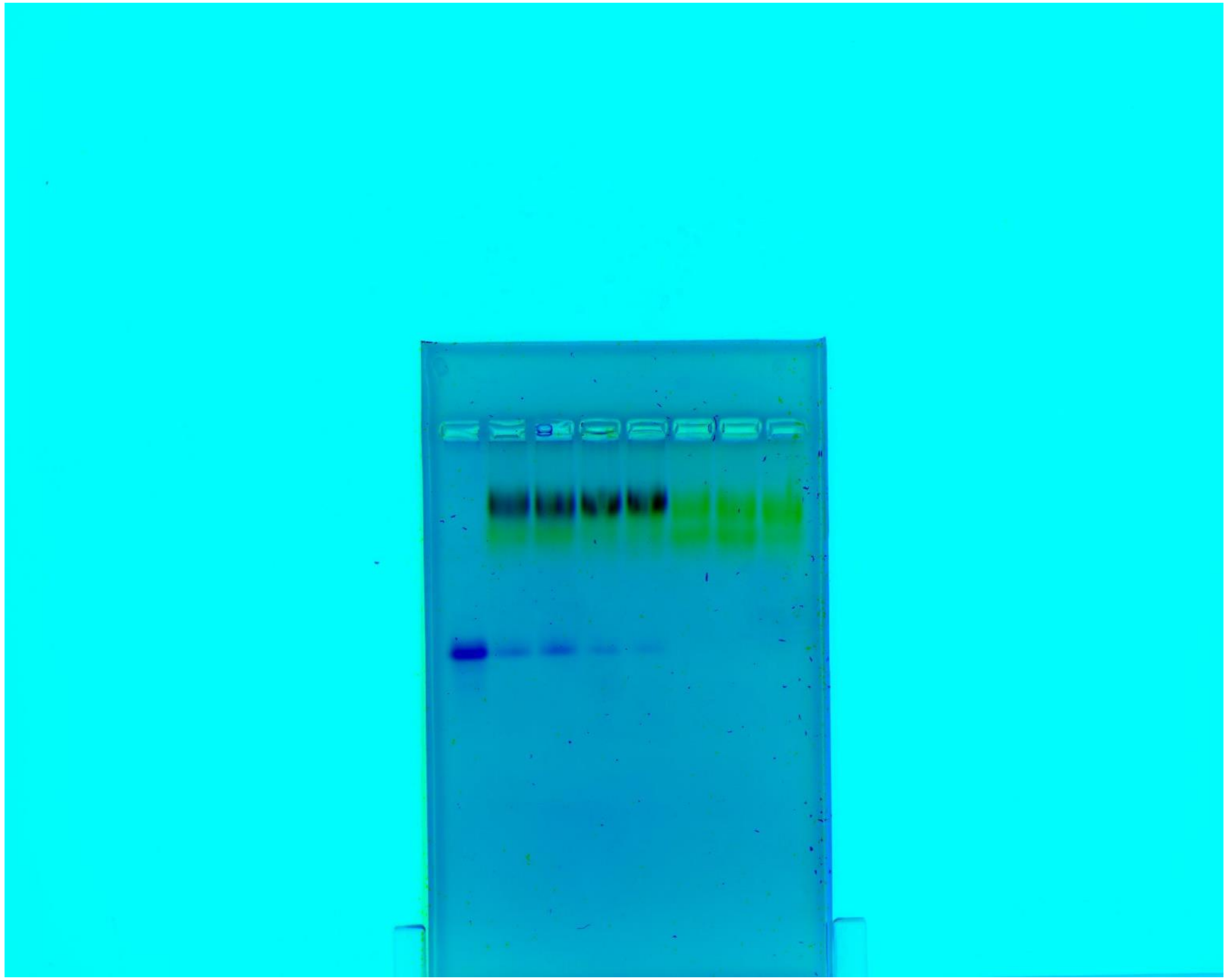

Lane assignment:
1 – RNA + 10 µM
2 – RNA + MDMX-S403D FL + 10 µM
3 – RNA + MDMX-S403D FL + 100 µM
4 – RNA + MDMX-S403D FL + 10 µM + EDTA
5 - RNA + MDMX-S403D FL + 100 µM + EDTA
6 – MDMX-S403D FL + 10 µM
7 - MDMX-S403D FL + 100 µM
8 - MDMX-S403D FL + 10 µM + EDTA

Raw data for Figure S5D

EMSA = Effect of TCEP and EDTA on the interaction between MDMX-S403D FL and mRNA *TP53* (GelRed staining)

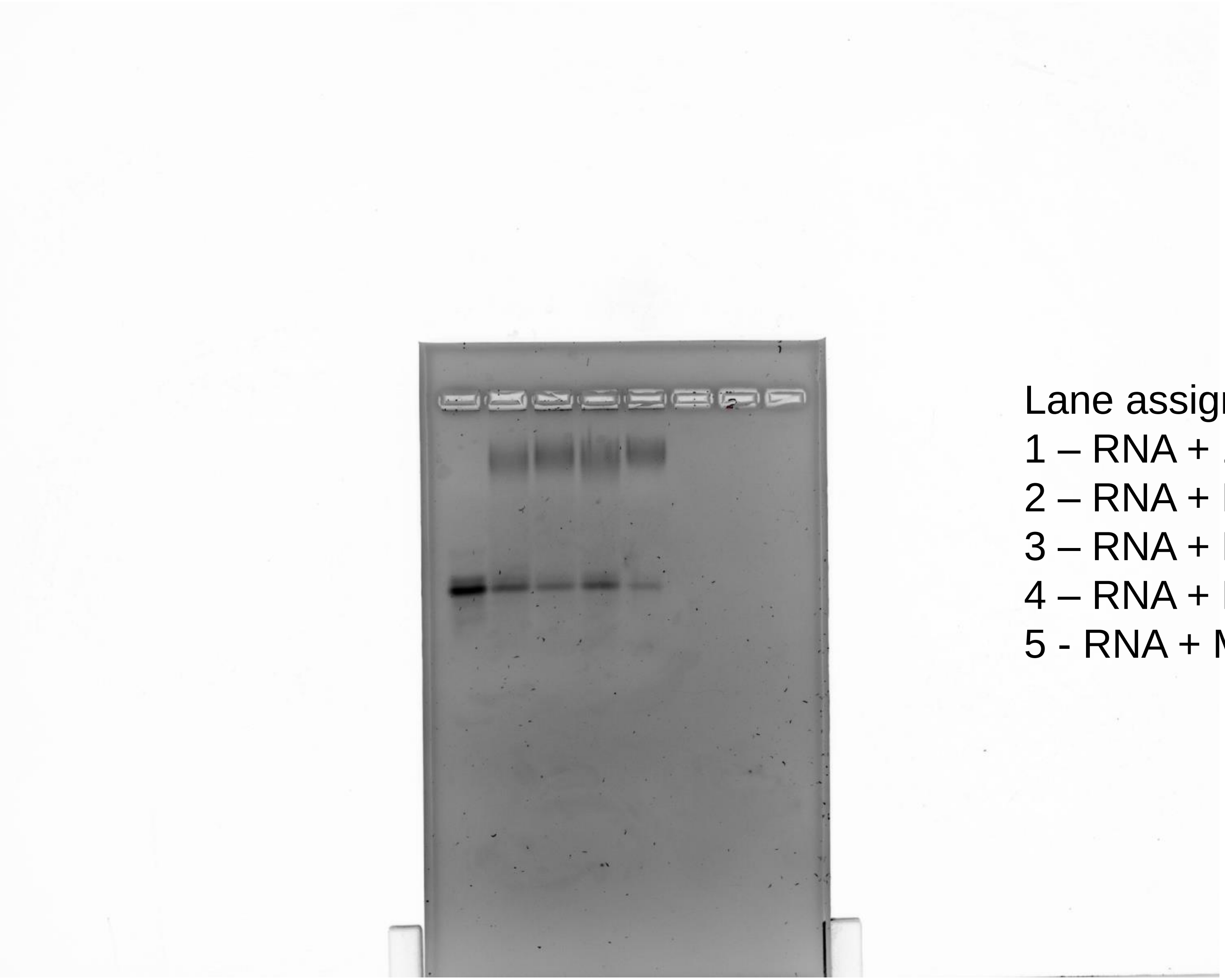

Lane assignment:
1 – RNA + 1 mM TCEP
2 – RNA + MDMX-S403D FL
3 – RNA + MDMX-S403D FL + 1 mM TCEP
4 – RNA + MDMX-S403D FL + EDTA
5 - RNA + MDMX-S403D FL + 1 mM TCEP + EDTA

Raw data for Figure S5D

EMSA = Effect of TCEP and EDTA on the interaction between MDMX-S403D FL and mRNA *TP53* (SYPRO Ruby st

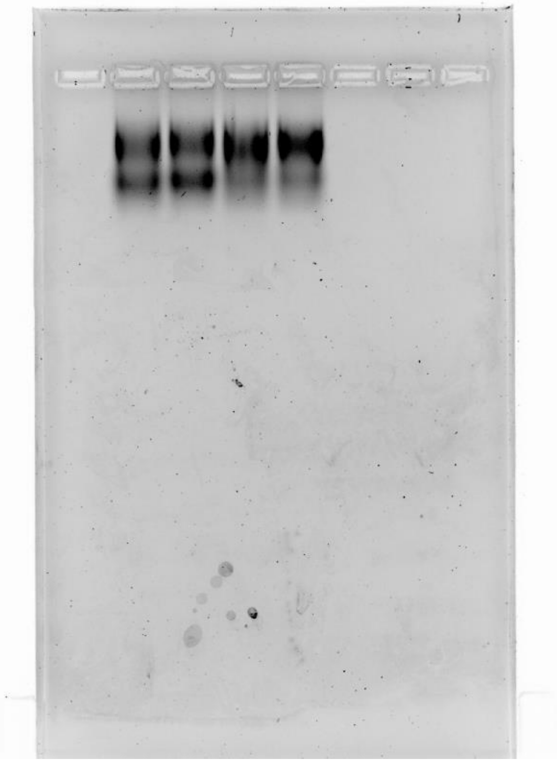

Lane assignment:
1 – RNA + 1 mM TCEP
2 – RNA + MDMX-S403D FL
3 – RNA + MDMX-S403D FL + 1 mM TCEP
4 – RNA + MDMX-S403D FL + EDTA
5 - RNA + MDMX-S403D FL + 1 mM TCEP + EDTA

Raw data for Figure S5D

EMSA = Effect of TCEP and EDTA on the interaction between MDMX-S403D FL and mRNA *TP53* (GelRed staining)

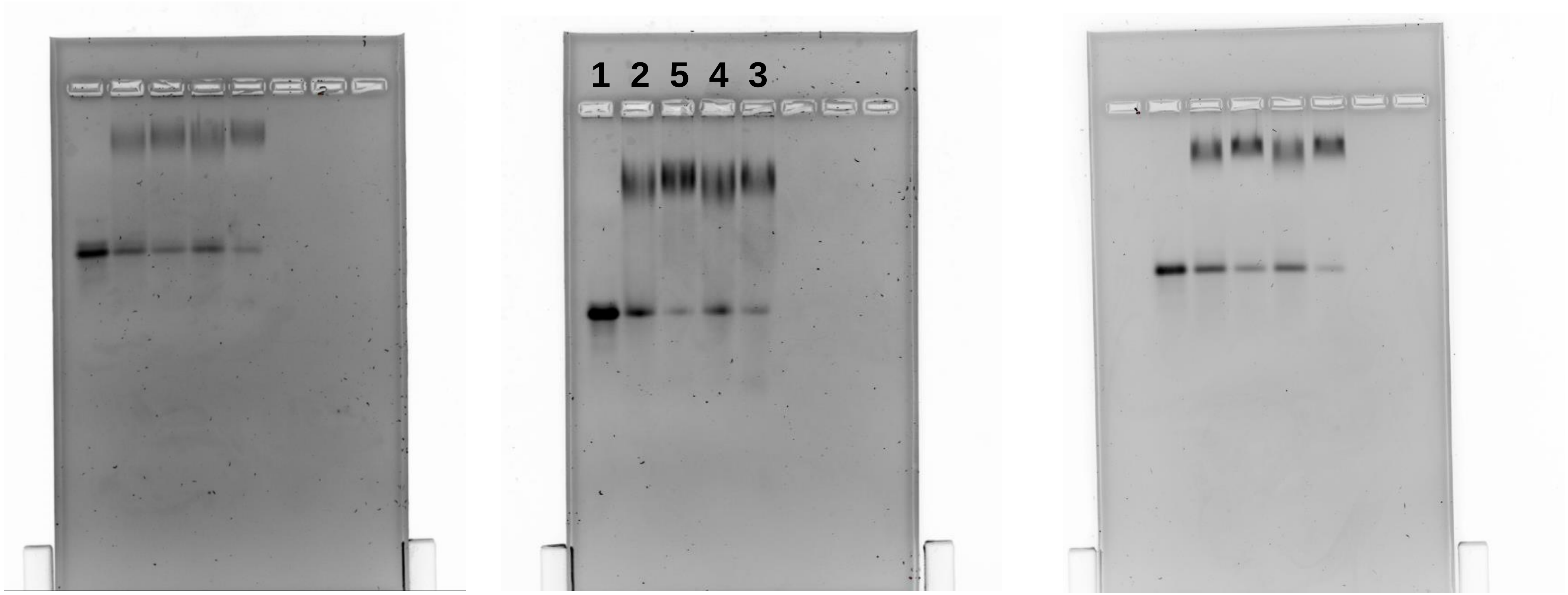

Lane assignment:
1 – RNA + 1 mM TCEP
2 – RNA + MDMX-S403D FL
3 – RNA + MDMX-S403D FL + 1 mM TCEP
4 – RNA + MDMX-S403D FL + EDTA
5 - RNA + MDMX-S403D FL + 1 mM TCEP + EDTA
